# Polysubstituted Pyrazoles as Corrosion Inhibitors for SAE 1020 Steel in 1 M HCl

**DOI:** 10.1021/acsomega.6c06392

**Published:** 2026-08-22

**Authors:** Gabriel R. Antunes, Ana J. F. Souza, Larissa L. Machado, Fillipe A. Carmo, Alfredo G. M. Castro, Denise C. Bertagnolli, Elivelton A. Ferreira, Julliane Yoneda, Diego Pereira Sangi

**Affiliations:** † Departamento de Química, Instituto de Ciências Exatas, 28110 Universidade Federal Fluminense, 27213-145 Volta Redonda, Rio de Janeiro, Brazil; ‡ Programa de Pós-graduação em Engenharia Metalúrgica, Universidade Federal Fluminense, 27255-125 Volta Redonda, Rio de Janeiro, Brazil

## Abstract

Most efficient corrosion inhibitors are heterocyclic compounds containing oxygen and nitrogen atoms, which act by adsorbing onto metal surfaces and donating electron pairs. Ketene dithioacetals are important building blocks in organic synthesis and have been widely used for the preparation of diverse heterocyclic compounds. In this work, ketene dithioacetals were used to synthesize polysubstituted pyrazoles that exhibited the ability to inhibit the corrosion of SAE 1020 carbon steel in 1 mol L^–1^ HCl. Theoretical calculations made it possible to correlate physicochemical properties with the different inhibition efficiencies observed experimentally and suggested that intramolecular hydrogen bonding may contribute to the differences in corrosion inhibition performance. The compound with the highest inhibition efficiency was further evaluated at different concentrations and maintained a corrosion inhibition efficiency above 90%, even at substantially lower inhibitor concentrations. Its adsorption behavior was well described by the Langmuir adsorption isotherm, indicating the coexistence of both physisorption and chemisorption on the SAE 1020 steel surface. These findings demonstrate that ketene dithioacetals provide an efficient synthetic route to functionalized pyrazoles with excellent potential as corrosion inhibitors for carbon steel in acidic media.

## Graphical abstract

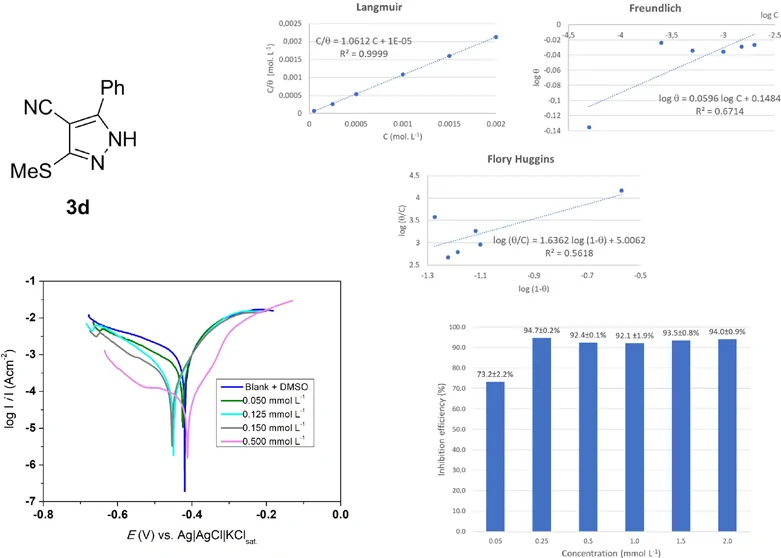

## 1 Introduction

Corrosion of metallic structures is a widespread phenomenon that has significant economic consequences and poses serious occupational and environmental risks due to equipment failure and the leakage of hazardous chemicals.^1–3^ One of the most effective approaches to mitigating corrosion is the use of corrosion inhibitors. Inorganic compounds such as chromates, phosphates, and molybdates have traditionally been used for this purpose. However, their high toxicity and associated environmental concerns have stimulated the search for safer alternatives.^3,4^

Organic corrosion inhibitors have been extensively investigated as one of the most practical and cost-effective strategies for protecting metallic materials exposed to aggressive environments, particularly acidic media used in industrial processes such as acid pickling, descaling, and oil well acidification. These compounds inhibit corrosion mainly through adsorption onto the metal surface, forming a protective barrier that suppresses both anodic metal dissolution and the cathodic hydrogen evolution reaction. Their inhibition efficiency is strongly influenced by the presence of heteroatoms, such as nitrogen, oxygen, sulfur, and phosphorus, as well as π-electron systems and functional groups capable of interacting with vacant metal orbitals. Consequently, considerable research efforts have focused on the design and synthesis of heterocyclic organic compounds with enhanced adsorption ability, improved inhibition efficiency, and lower environmental impact.^3–8^

Among these compounds, azole derivatives, such as oxadiazoles, triazoles, and pyrazoles, have attracted considerable attention because of their structural versatility and remarkable corrosion inhibition performance. These nitrogen-containing heterocyclic compounds possess lone-pair electrons and conjugated π-systems that facilitate strong adsorption onto metal surfaces, making them particularly effective corrosion inhibitors.^9–11^

For instance, substituted 1,3,4-oxadiazoles have been reported as effective corrosion inhibitors for mild steel in hydrochloric acid solutions, with their performance being strongly influenced by molecular structure and the nature of the substituents.^12^ Likewise, 1,2,4-triazole derivatives have demonstrated significant corrosion inhibition performance for mild steel in acidic environments, where adsorption onto the metal surface plays a crucial role in the protection mechanism.^13^

Furthermore, electrochemical and quantum chemical studies have confirmed that electron-rich heterocyclic systems enhance metal–inhibitor interactions and improve the corrosion protection afforded by triazole-based compounds for aluminum alloys in hydrochloric acid solutions.^14–16^

Pyrazole and its derivatives have attracted considerable attention because of their strong coordination ability and excellent corrosion inhibition properties. The nitrogen atoms and delocalized π-electrons of the pyrazole ring promote effective adsorption onto metal surfaces, resulting in the formation of protective hydrophobic layers that mitigate corrosion. The presence of two adjacent nitrogen atoms makes the ring electron-rich, favoring strong coordination with metal atoms and enhancing its ability to act as a ligand through π-electron interactions. These compounds can function as both electron donors and electron acceptors, enabling strong adsorption onto metallic substrates. Their inhibition efficiency is influenced by both electronic effects and the spatial arrangement of substituent groups, which affect molecular adsorption, surface coverage, and the stability of the protective film.^17–23^

Ketene dithioacetals are versatile building blocks in the synthesis of heterocyclic compounds with potential applications in biological and materials sciences. Recently, we employed the double vinylic substitution of methylsulfanyl groups in ketene dithioacetals bearing electron-withdrawing groups to synthesize heterocyclic compounds such as oxazolidines, imidazolidines, oxazinanes, hexahydropyrimidines, and diazepines, which exhibited promising corrosion inhibition properties.^24–29^

The widespread application of pyrazole derivatives as corrosion inhibitors motivated us to investigate their facile synthesis from ketene dithioacetals. In this work, polysubstituted pyrazoles were synthesized from ketene dithioacetals through sequential vinylic substitution and cyclization reactions. Their corrosion inhibition performance toward SAE 1020 carbon steel was evaluated by gravimetric measurements, and theoretical calculations were performed to rationalize the experimentally observed inhibition efficiencies.

## 2 Materials and Methods

### 2.1 General Information

Chemicals were purchased from commercial suppliers and used without further purification. Cyano ketene dithioacetals (**1a**–**c**) were prepared by a one-pot, three-step synthetic procedure as previously described.^30^

Microwave-assisted reactions were carried out using an Anton Paar Monowave 300 reactor. Thin-layer chromatography (TLC) analyses were performed on commercial aluminum-backed silica gel plates (0.2 mm, Macherey-Nagel), and the chromatograms were visualized under ultraviolet light (254 nm).

Melting points were determined using a Gehaka PF1500 Farma melting point apparatus. Infrared (IR) spectra were recorded on a PerkinElmer Spectrum 100 spectrophotometer equipped with an attenuated total reflectance (ATR) accessory. Nuclear magnetic resonance (NMR) spectra were acquired on Bruker Avance DRX 400 and Varian VNMRS 500 MHz spectrometers. Mass spectrometric (MS) analyses were performed using a Shimadzu GCMS-QP2010 Plus and a Waters/Micromass UPLC-QTof-MS system, providing a mass resolution greater than 10,000 FWHM.

### 2.2 General Procedure for the Synthesis of Polysubstituted Pyrazoles

Polarized dithioacetal (**1a**–**c**) (1 mmol), hydrazine derivative (**2a**–**e**) (1 mmol), and ethanol (3 mL) were added to a microwave reaction vial. The reaction mixture was irradiated for 60 min at 110 °C under continuous stirring.

After completion of the reaction, the solvent was removed under reduced pressure, and the crude products were purified by column chromatography to afford compounds **3a**–**d** and **4a**-**e** in greater than 99% purity.

The synthesized compounds were characterized by infrared spectroscopy, mass spectrometry, and proton (^1^H) and carbon (^13^C) nuclear magnetic resonance spectroscopy. The corresponding spectra (Figures S1–S24) are provided in the Supporting Information.

#### 5-Amino-4-cyano-3-(methylthio)-1*H*-pyrazole-1-carbothioamide (**3a**)

Yield 79%. ^1^H NMR (400 MHz, DMSO) δ 9.85 (s, 1H), 9.24 (s, 1H), 8.87 (s, 2H), 2.57 (s, 3H). ^13^C NMR (101 MHz, DMSO) δ 176.52, 156.59, 151.52, 113.41, 73.51, 13.06.

#### 5-Amino-3-(methylthio)-1-phenyl-1*H*-pyrazole-4-carbonitrile (**3b**)

Yield 88%. ^1^H NMR (400 MHz, MeOD) δ 7.57–7.42 (m, 5H), 2.53 (s, 3H). ^13^C NMR (101 MHz, MeOD) δ 152.58, 150.24, 137.26, 129.38, 128.27, 124.39, 113.36, 74.16, 12.91. MS (*m*/*z*, (%)): 230(100); 197(70); 77(67); 51(23).

#### 5-Amino-3-(methylthio)-1*H*-pyrazole-4-carbonitrile (**3c**)

Yield 87%. ^1^H NMR ^13^C NMR (101 MHz, DMSO) δ ^1^H NMR (400 MHz, DMSO) δ 11.96 (s, 1H), 6.45 (s, 2H), 2.43 (s, 3H).^13^C NMR (101 MHz, DMSO) δ 154.60, 148.16, 115.18, 72.99, 14.17. MS (*m*/*z*, (%)): 154(100); 121(38); 67(20); 45(25).

#### 3-(Methylthio)-5-phenyl-1*H*-pyrazole-4-carbonitrile (**3d**)

Yield 65%. ^1^H NMR (400 MHz, DMSO-*d*_6_) δ 14.15 (s, 1H), 7.85–7.79 (m, 2H), 7.56 (dt, *J* = 13.3, 6.9 Hz, 3H), 2.62 (s, 3H). IR (ATR) (*v*_max_/cm^–1^): 3208, 2227, 1561, 1488, 1072, 964, 769. HRMS (ESI): Calcd for C_11_H_10_N_3_S^+^ [M + H]^+^ = 216.0597, found 216.0586.

#### Methyl 5-Amino-3-(methylthio)-1*H*-pyrazole-4-carboxylate (**4a**)

Yield 73%. ^1^H NMR (400 MHz, DMSO) δ 11.78 (s, 1H), 6.05 (s, 2H), 3.67 (s, 3H), 2.35 (s, 3H). ^13^C NMR (101 MHz, DMSO) δ 164.07, 153.34, 114.39, 91.89, 50.69, 12.84.

#### (5-Amino-3-(methylthio)-1-phenyl-1*H*-pyrazol-4-yl)­(phenyl)­methanone (**4b**)

Yield 91%. ^1^H NMR (400 MHz, DMSO) δ 7.61–7.42 (m, 10H), 7.05 (s, 2H), 2.30 (s, 3H). ^13^C NMR (101 MHz, DMSO) δ 190.15, 152.41, 148.88, 140.87, 137.77, 131.21, 130.00, 128.62, 128.12, 127.72, 124.18, 103.54, 13.87. MS (*m*/*z*, (%)): 309(97); 308(66); 105(510); 77(100).

#### Methyl 5-Amino-3-(methylthio)-1-phenyl-1*H*-pyrazole-4-carboxylate (**4c**)

Yield 81%. ^1^H NMR (400 MHz, DMSO) δ 7.57–7.52 (m, 4H), 7.44–7.40 (m, 1H), 6.41 (s, 2H), 3.74 (s, 3H), 2.40 (s, 3H). ^13^C NMR (101 MHz, DMSO) δ 164.00, 151.44, 149.26, 138.19, 129.92, 127.92, 124.15, 92.88, 51.06, 12.75. MS (*m*/*z*, (%)): 263(100); 231(72); 198(44); 77(56).

#### Methyl 5-Amino-3-(methylthio)-1-(4-nitrophenyl)-1*H*-pyrazole-4-carboxylate (**4d**)

^1^H NMR (400 MHz, CDCl_3_) δ 8.37 (d, *J* = 8.7 Hz, 2H), 7.82 (d, *J* = 8.8 Hz, 2H), 5.58 (s, 2H), 3.88 (s, 3H), 2.54 (s, 3H). ^13^C NMR (101 MHz, CDCl_3_) δ 164.64, 151.70, 151.07, 145.81, 143.04, 125.41, 122.44, 95.28, 51.24, 13.09.

#### (5-Amino-3-(methylthio)-1-tosyl-1*H*-pyrazol-4-yl)­(phenyl)­methanone (**4e**)

^1^H NMR (400 MHz, CDCl_3_) δ 7.92 (d, *J* = 8.4 Hz, 2H), 7.54–7.50 (m, 3H), 7.45–7.41 (m, 7.0 Hz, 2H), 7.38 (d, *J* = 8.0 Hz, 2H), 7.33 (s, 2H), 2.47 (s, 3H), 2.32 (s, 3H). ^13^C NMR (101 MHz, CDCl_3_) δ 191.23, 155.28, 154.56, 146.22, 139.65, 133.74, 131.30, 130.00, 128.16, 128.07, 127.55, 102.79, 21.78, 14.11. IR (ATR) (*v*_max_/cm^–1^): 3448, 3311, 1609, 1591, 1493, 1486, 1369, 1305, 1171, 918, 703. HRMS (ESI): Calcd for C_18_H_18_N_3_O_3_S_2_^+^ [M + H]^+^ = 388.0791, found 388.0753.

### 2.3 Computational Details

Certain theoretical parameters derived from Density Functional Theory (DFT) can be correlated with the corrosion inhibition efficiency of a compound.^31^ To obtain these parameters, the molecular structures of the studied compounds were optimized at the B3LYP/6–311++G­(d,p) level of theory using the Gaussian 16W software package.^32^

The energies of the highest occupied molecular orbital (*E*_HOMO_) and the lowest unoccupied molecular orbital (*E*_LUMO_) were calculated from the optimized structures. These energies are related to the ionization potential (*I*) and electron affinity (*A*) as defined by eqs 1 and 2.^31^

1 $$I=-E_{\mathrm{HOMO}}$$

2 $$A=-E_{\mathrm{LUMO}}$$

Additional physicochemical parameters associated with corrosion inhibition, including hardness (η) and softness (σ), were calculated from the ionization potential and electron affinity according to eqs 3 and 4.^31^

3 η=(I−A)/2

4 σ=1/η

### 2.4 Mass Loss Measurements

SAE 1020 carbon steel plates with the following composition (wt %): C (0.18–0.23), Mn (0.30–0.60), S (0.05), and P (0.03), and dimensions of 2.5 × 2.5 × 0.13 cm^3^, were mechanically polished using successive grades of emery paper (150, 220, 320, 400, and 600 grit). After polishing, the specimens were thoroughly rinsed with deionized (Milli-Q) water.

The blank solution consisted of 5% (v/v) dimethyl sulfoxide (DMSO) in 1.0 mol L^–1^ HCl. Inhibitor solutions were prepared under the same conditions by dissolving each inhibitor at a concentration of 2.0 mmol L^–1^ in the blank solution. The steel specimens were immersed in the test solutions for 4 h. The compound exhibiting the highest inhibition efficiency was further evaluated at concentrations of 0.05, 0.25, 0.50, 1.00, and 1.50 mmol L^–1^ under identical experimental conditions. All experiments were performed in triplicate.

Corrosion resistance was evaluated by the gravimetric method through determination of the mass loss of the steel specimens after immersion in the acidic solution. The mass loss (*w*) was calculated from the difference between the initial mass (*m_i_*) and the final mass (*m_f_*) of each specimen, as expressed in eq 5. The inhibition efficiency (IE) was then calculated using eq 6, where *w* ^0^ and *w* are the mass losses measured in the absence and presence of the inhibitor, respectively.

5 $$w=m_{f}-m_{i}$$

6 $$\mathrm{IE}\left(\%\right)=\frac{\left(w^{0}-w\right)}{w^{0}}.100$$

### 2.5 Adsorption Isotherms

The mass loss measurements were used to determine the surface coverage (θ), corresponding to the fraction of the metal surface covered by adsorbed inhibitor molecules, according to eq 7.

7 $$\theta =\frac{\left(w^{0}-w\right)}{w^{0}}$$

The surface coverage values were analyzed using the Langmuir, Freundlich, and Flory–Huggins adsorption isotherms (eqs 8–10).^33^

8 $$\frac{C}{\theta }=\frac{1}{K_{\mathrm{ads}}}+C$$

9 $$\log\theta =\log K_{f}+\frac{1}{n}\log C$$

10 $$\log\left(\frac{\theta }{C}\right)=\log K_{\mathrm{ads}}+F_{H}\log\left(1-\theta \right)$$

where *C* is the inhibitor concentration, θ the is the surface coverage or fraction of adsorbed molecules, *K*_ads_ is the adsorption constant, *K*_*f*_ and *n* are the Freundlich constants and F_H_ is the Flory–Huggins interaction parameter.

The adsorption mechanism was further evaluated by calculating the standard Gibbs free energy of adsorption (Δ*G*°_ads_) using eq 11.^33^

11 $${\Delta G^{{}^{\circ}}}_{\mathrm{ads}}=-\mathrm{RT}\ln\left(C_{\mathrm{water}}K_{\mathrm{ads}}\right)$$

where *R* is the universal gas constant (8.314 J mol^–1^ K^–1^), *T* is the absolute temperature and *C*_water_ is the molar water concentration of 55.5.

### 2.6 Electrochemical Measurements

The electrolyte solutions consisted of 5% (v/v) DMSO in 1.0 mol L^–1^ HCl in the absence or presence of the inhibitor at concentrations of 0.05, 0.125, 0.150, and 0.50 mmol L^–1^. SAE 1020 carbon steel specimens were immersed in the test solutions for 30 min at 25 °C under open-circuit potential (OCP) conditions. Potentiodynamic polarization measurements were subsequently performed using a VersaSTAT 3 potentiostat (AMETEK Scientific Instruments). At least two independent measurements were carried out for each condition. Polarization curves were recorded by scanning the potential from −150 mV to +150 mV relative to the OCP at a scan rate of 0.166 mV s^–1^.^34,35^

### 2.7 Scanning Electron Microscopy (SEM)

The surface morphology of the steel specimens was examined by scanning electron microscopy (SEM) using an FEI Quanta 3D FEG microscope.^27^

## 3 Results and Discussion

Using ketene dithioacetals (**1a**-**c**) as starting materials, we carried out a microwave-assisted synthesis employing hydrazine derivatives as nucleophiles to obtain polysubstituted pyrazoles. The synthesized compounds were subsequently evaluated as corrosion inhibitors to investigate their potential for protecting carbon steel against corrosion-induced mass loss. Nine pyrazoles (**3a**–**d** and **4a**–**e**) were obtained in yields ranging from 43% to 91% (Figure 1).

**Figure 1:**
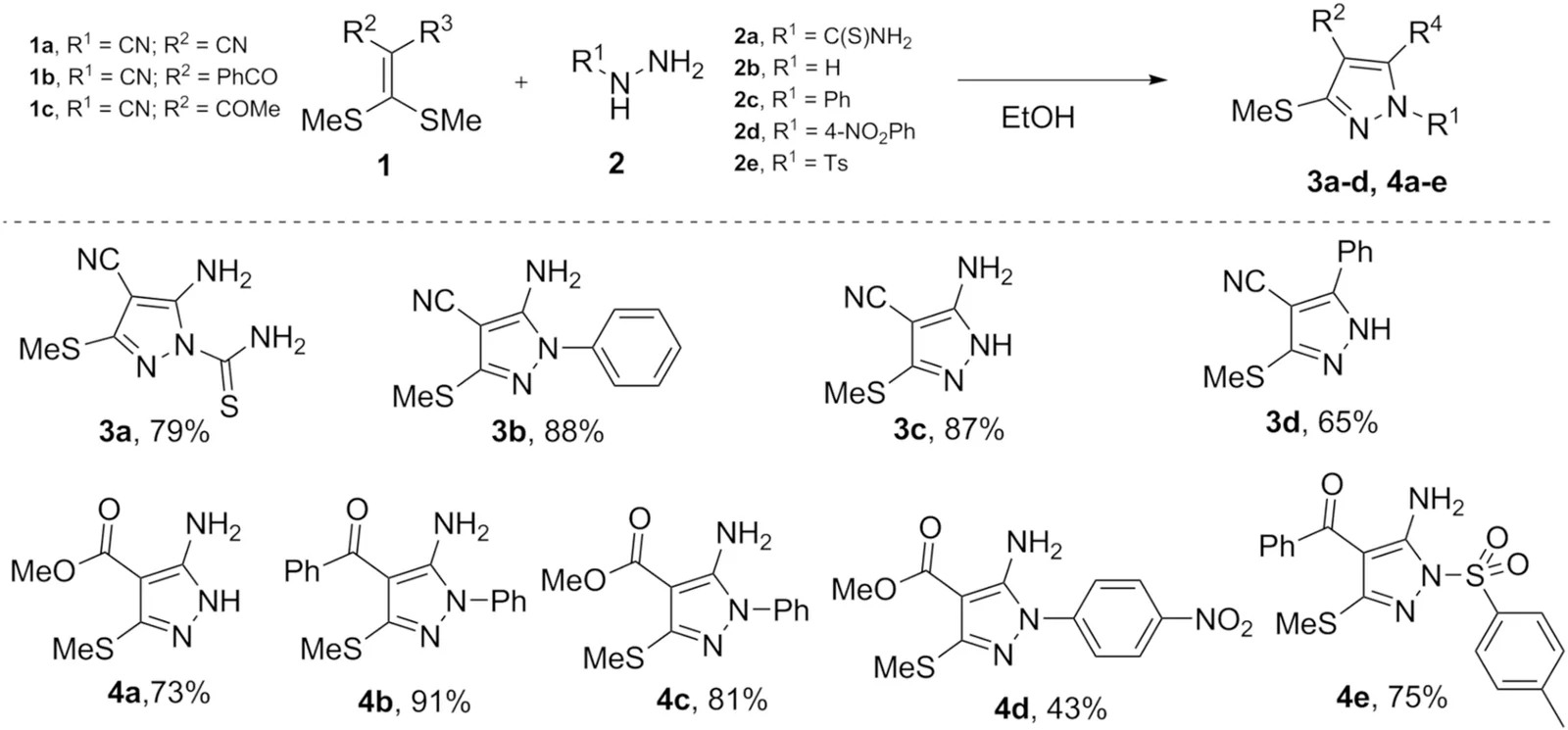
Synthesis of polysubstituted pyrazoles (**3a**–**d**, **4a**–**e**) with respective yields.

All synthesized compounds were characterized by ^1^H and ^13^C NMR spectroscopy. The ^1^H NMR spectra exhibited singlets in the range of 2.30–2.62 ppm, integrating for three protons and assigned to the methylsulfanyl (SCH_3_) groups. In addition, pyrazoles **3a**, **3c**, and **4a**–**e** displayed singlets integrating for two protons, corresponding to the amino (NH_2_) groups. The NH_2_ signal was not observed in the spectrum of compound **3b** because the use of MeOD as the solvent promotes rapid proton exchange.

The ^1^H NMR spectra of compounds **4a**, **4c** and **4d** exhibited additional singlets at 3.64, 3.74, and 3.88 ppm, respectively, integrating for three protons and assigned to the methoxy (OCH_3_) substituents. In contrast, compounds **4b** and **4e** displayed signals in the range of 7.30–7.90 ppm, corresponding to aromatic protons.

The ^13^C NMR spectra of all pyrazole derivatives exhibited signals assigned to the methylsulfanyl carbon atoms in the range of 12–14 ppm. Furthermore, compounds **3a–d** showed characteristic signals between 113 and 115 ppm, attributed to the nitrile carbon. Compounds **4a**, **4c** and **4d** exhibited signals at approximately 164 ppm, corresponding to the ester carbonyl carbon, whereas compounds **4b** and **4e** showed signals at 190 and 191 ppm, respectively, assigned to the ketone carbonyl carbon.

The corrosion inhibition performance of pyrazoles **3a**–**d** and **4a**–**e** was evaluated by gravimetric measurements in 1.0 mol L^–1^ HCl. This highly aggressive medium was selected to accelerate the corrosion process and reduce the experimental time, while also simulating acidic industrial processes such as pickling and descaling, in which acid solutions are used to remove oxide layers from metallic surfaces and corrosion rates are particularly high.^26,27^

Compounds **3a–d** exhibited inhibition efficiencies higher than 75%, whereas compounds **4a–e** showed inhibition efficiencies below 41%. Interestingly, all of the most effective inhibitors contained a cyano group in their molecular structures (Table 1). Since compound **3d** exhibited the highest inhibition efficiency, its performance was further evaluated at lower concentrations. Remarkably, inhibition efficiencies above 90% were maintained even at a concentration of 0.25 mmol L^–1^, as shown in Figure 2.

**Figure 2:**
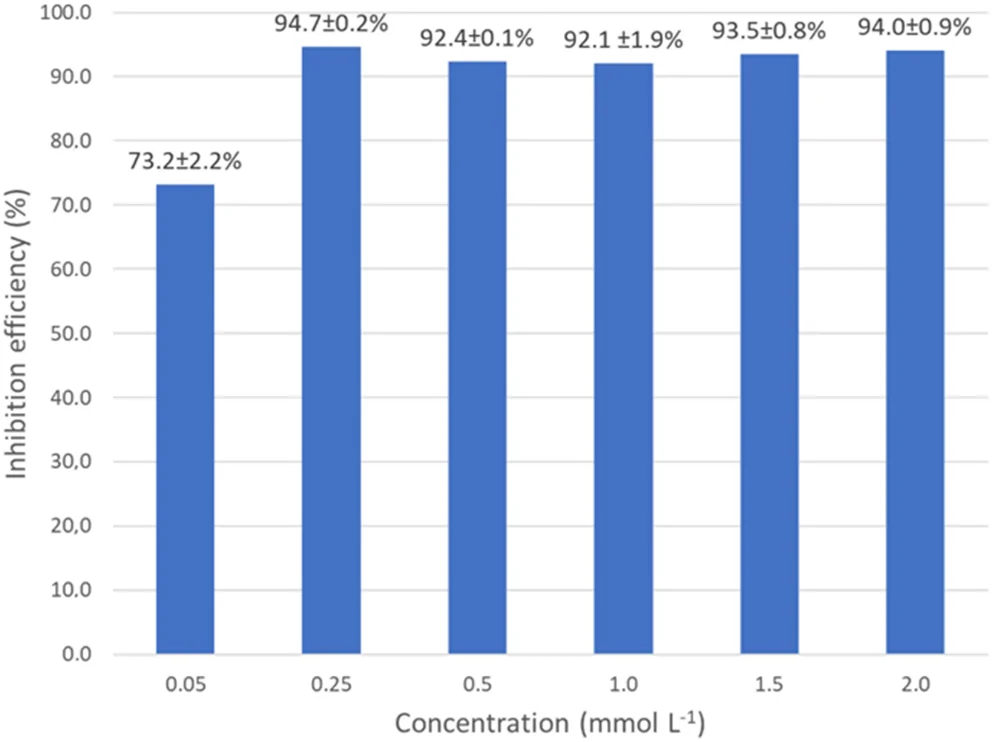
Inhibition efficiency of compound **3d** at different concentrations.

**Table 1:** Inhibition Efficiency of Synthesized Pyrazole Derivatives

Inhibitor	IE (%)^a^
**3a**	74.6 ± 4.1
**3b**	83.0 ± 1.0
**3c**	85.5 ± 1.4
**3d**	93.9 ± 0.9
**4a**	13.9 ± 4.6
**4b**	24.2 ± 5.0
**4c**	22.6 ± 1.9
**4d**	33.3 ± 1.9
**4e**	40.7 ± 2.5

a Relative to mass loss of the solution (5% v/v in HCl 1M) without inhibitor.

Figure 3a shows the polarization curves (PCs) obtained for SAE 1020 steel in 1.0 mol L^–1^ HCl containing different concentrations of compound **3d**. As the inhibitor concentration increased up to 0.150 mmol L^–1^, a progressive decrease in the cathodic current density was observed. In contrast, no significant changes were detected in the anodic branch within this concentration range. These results indicate that corrosion inhibition was predominantly under cathodic control at inhibitor concentrations up to 0.150 mmol L^–1^.

**Figure 3:**
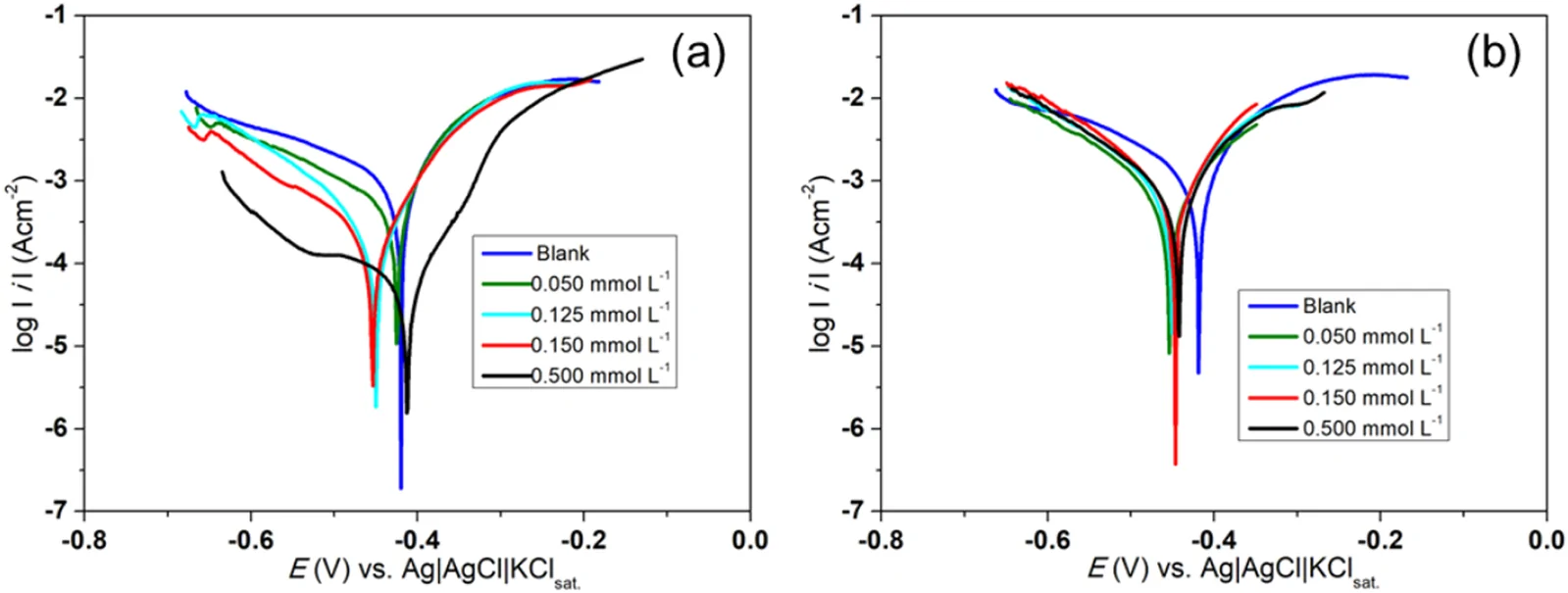
PC curves for SAE 1020 steel in 1.0 mol L^–1^ HCl solution in the absence and presence of different concentrations of compounds **3d** (a) and **4a** (b).

At an inhibitor concentration of 0.50 mmol L^–1^, the polarization behavior changed to mixed-type inhibition, with reductions observed in both the cathodic and anodic current densities. The variations in the open-circuit potential (OCP) were negligible and remained within the experimental error.^36^

On the other hand, the polarization curves obtained in solutions containing compound **4a** show only a slight decrease in the OCP (Figure 3b). Moreover, no significant differences are observed among the polarization curves of steel immersed in solutions containing different concentrations of compound **4a**. These results are consistent with those obtained from the mass loss measurements.

Figure 4 presents SEM micrographs of SAE 1020 steel before and after immersion for 4 h in 1.0 mol L^–1^ HCl in the absence and presence of inhibitors **4a** and **3d** at a concentration of 2.0 mmol L^–1^.

**Figure 4:**
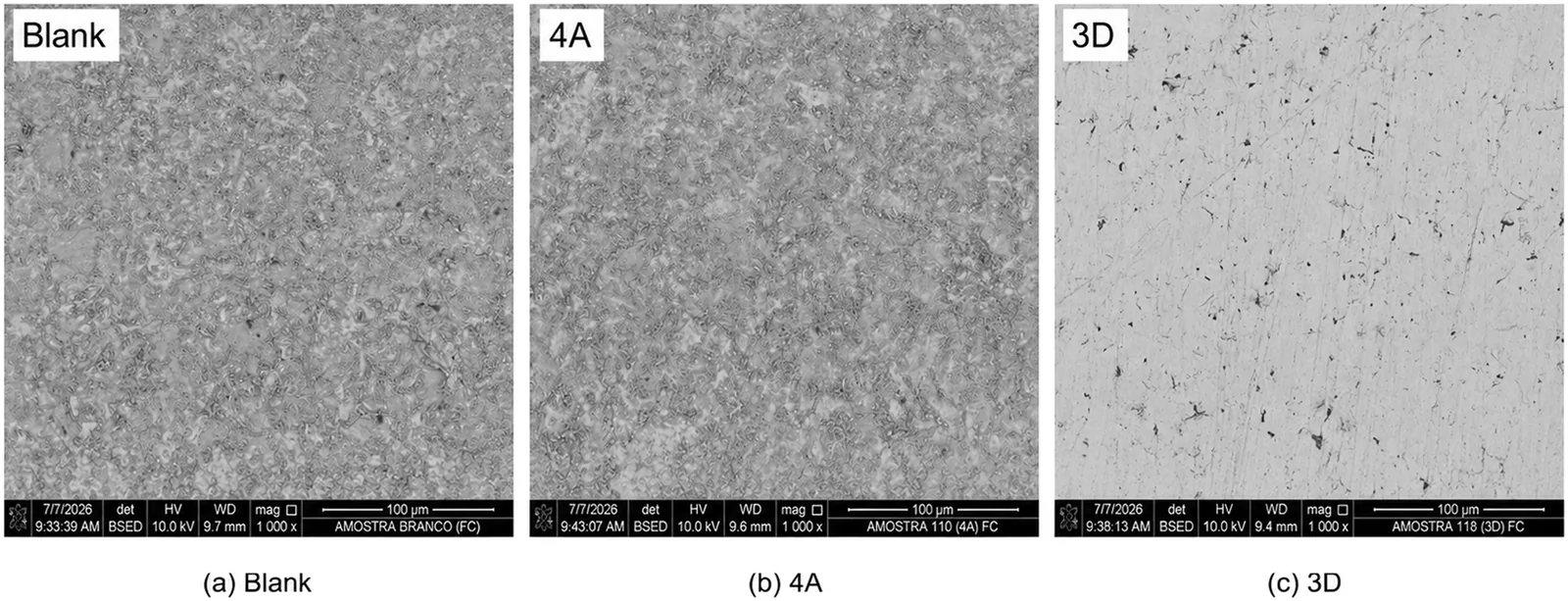
SEM micrographs of the SAE 1020 steel surface after immersion for 4 h in 1.0 mol L^–1^ HCl solution in the absence of inhibitor (a) and in the presence of inhibitors **4a** (b) and **3d** (c) at a concentration of 2.0 mmol L^–1^.

The SEM micrographs of the samples immersed in the blank solution and in the solution containing inhibitor **4a** (Figure 4a,b) show the presence of corrosion products, possibly resulting from oxidation of the steel after its removal from the acidic solution and subsequent exposure to the atmosphere. In contrast, the SEM micrograph of the sample immersed in the solution containing inhibitor **3d** (Figure 4c) showed no evidence of corrosion products, demonstrating its high corrosion inhibition efficiency.

Theoretical calculations can provide valuable insights into the interpretation of experimental results. Density functional theory (DFT) has been widely employed to explain the corrosion inhibition performance of organic molecules because it provides molecular-level information on their electronic structure, reactivity, and adsorption capability on metallic surfaces.^37–39^

Organic compounds containing polar functional groups are adsorbed onto metal surfaces through donor–acceptor interactions between heteroatoms in the inhibitor molecules and metal atoms. In these interactions, the inhibitor acts as a Lewis base by donating electron density, whereas the metal behaves as a Lewis acid. Consequently, the stability of the adsorbed layer can be interpreted according to Pearson’s Hard and Soft Acids and Bases (HSAB) principle. Since metallic surfaces are generally considered soft acids, inhibitors with soft-base character are expected to exhibit stronger adsorption and, consequently, higher corrosion inhibition efficiencies in acidic media.^40^

According to this concept, a smaller highest occupied molecular orbital–lowest-unoccupied molecular orbital (HOMO–LUMO) energy gap (Δ*E*) is generally associated with higher molecular reactivity and a greater tendency for charge transfer between the inhibitor and the metal surface, thereby favoring adsorption. Accordingly, compounds with lower Δ*E* values are often expected to exhibit higher corrosion inhibition efficiencies. Besides the HOMO–LUMO energy gap, the frontier orbital energies provide information about the adsorption mechanism. Higher HOMO energies favor electron donation to the vacant d orbitals of iron, whereas lower LUMO energies facilitate electron acceptance through back-donation. Likewise, lower hardness and higher softness indicate greater molecular polarizability and easier electron density redistribution during adsorption, strengthening donor–acceptor interactions with the steel surface according to the HSAB principle.^37,41,42^

The calculated physicochemical parameters of the studied compounds are summarized in Table 2.

**Table 2:** Physicochemical Properties Calculated for Compounds **3a**–**d** and **4a**–**e**

Compound	*E*_HOMO_ (eV)	*E*_LUMO_ (eV)	Δ*E* (eV)	η	σ
**3a**	–6.62	–2.01	4.61	2.31	0.43
**3b**	–6.13	–1.36	4.77	2.39	0.42
**3c**	–6.41	–1.01	5.41	2.70	0.37
**3d**	–6.32	–1.85	4.47	2.24	0.45
**4a**	–5.81	–0.64	5.17	2.58	0.39
**4b**	–5.88	–1.61	4.27	2.13	0.47
**4c**	–5.80	–1.12	4.68	2.34	0.43
**4d**	–6.18	–1.94	4.25	2.12	0.47
**4e**	–6.25	–3.02	3.23	1.62	0.62

Analysis of the **3a–d** series (Figure 2) shows that all compounds contain a cyano group at the R2 position, while the substituents at the R1 and R4 positions vary. Compounds **3a** and **3d** exhibited the lowest HOMO–LUMO energy gaps and hardness values, whereas compounds **3b** and **3c** showed the highest values (Table 2). These results are consistent with the superior inhibition performance of compound **3d** (Table 1) and the slightly lower efficiencies observed for compounds **3b** and **3c**.

The molecular electrostatic potential maps complement the global quantum descriptors by identifying electron-rich regions that are more likely to interact with the steel surface. Therefore, the MEP analysis helps to identify the preferential adsorption sites and to rationalize differences in inhibition efficiency among structurally related compounds.^37,38^

Although the calculated physicochemical parameters suggest that compound **3a** should exhibit higher inhibition efficiency than that observed experimentally, this behavior may be attributed to the electron-withdrawing substituent at the R1 position. As illustrated by the electrostatic potential maps (Figure 5), the R1 substituent in compound **3a** decreases the electron density over the pyrazole ring, reducing the availability of electron density for interaction with the metal surface. Consequently, the adsorption of compound **3a** onto the steel surface is expected to be less favorable, leading to a lower inhibition efficiency than predicted solely from its frontier molecular orbital parameters.

**Figure 5:**
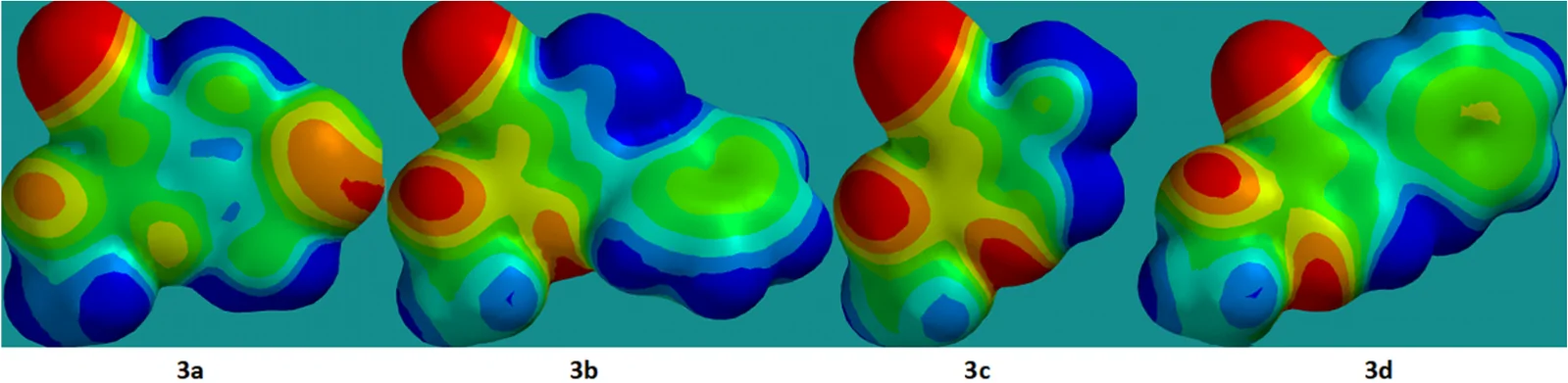
Electrostatic potential maps for compounds **3a**–**d** in the energy range from −100.00 kJ/mol (red) to +100.00 kJ/mol (blue).

Besides affecting the molecular geometry, the formation of intramolecular hydrogen bonds can modify the electron density distribution by engaging lone-pair electrons in these interactions, thereby reducing their availability for intermolecular interactions and adsorption onto the metal surface.

Concerning the **4a–e** series, although their physicochemical parameters are not markedly different from those calculated for the **3a–d** series, their inhibition efficiency decreases significantly. It is noteworthy that the main structural difference between the two series is the substituent at the R2 position. While compounds in the **3a–d** series contain a cyano group, those in the **4a–e** series contain a carbonyl group. The carbonyl oxygen can form an intramolecular hydrogen bond^43^ with one of the hydrogen atoms of the amino group at the R4 position, resulting in the formation of a six-membered pseudoring (Figure 6).

**Figure 6:**
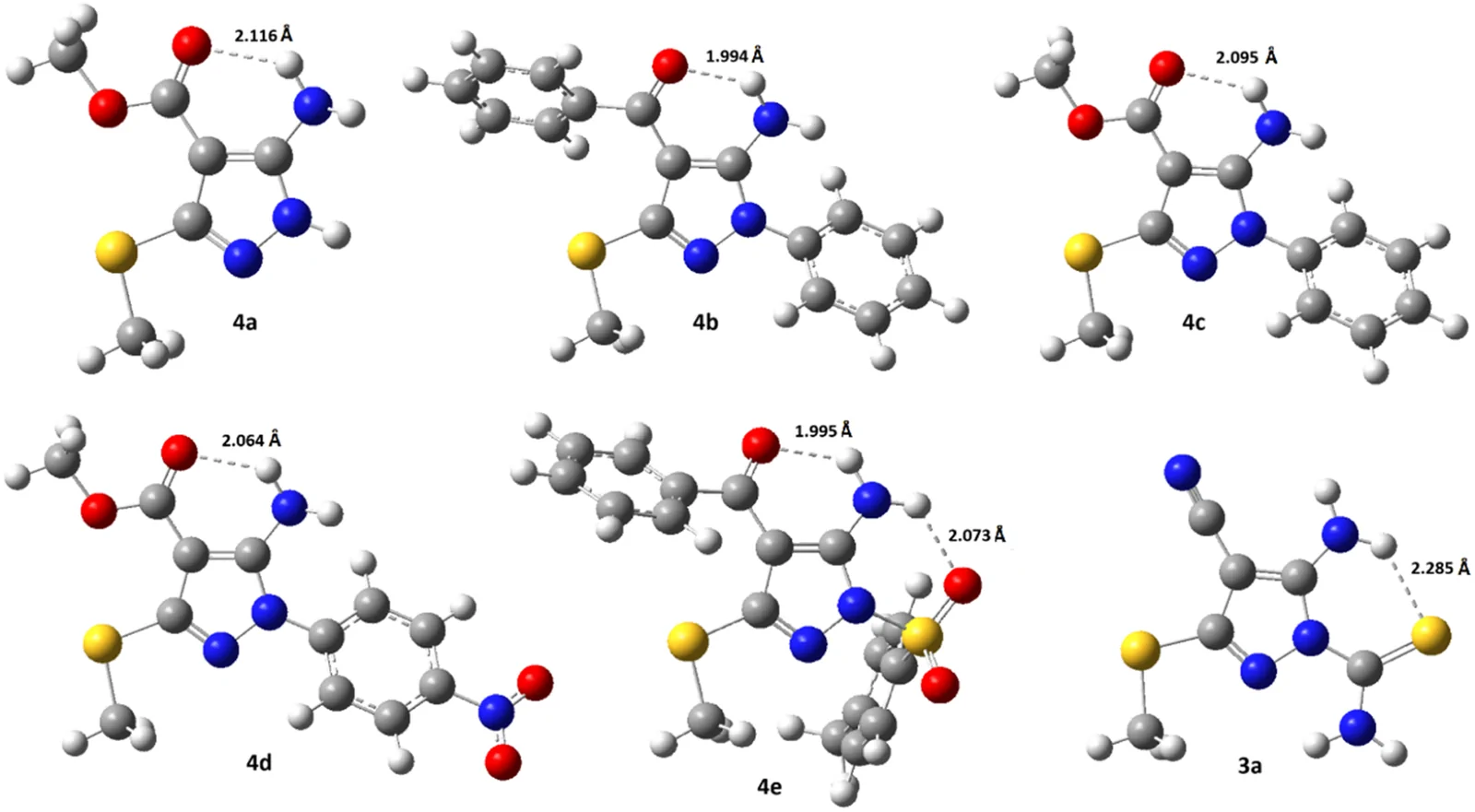
Intramolecular hydrogen bonds in compounds **4a**–**e** and **3a**.

Saha and co-workers^44^ reported that, under chemisorption conditions, intramolecular hydrogen bonding plays an important role by preventing chain propagation between neighboring inhibitor molecules, causing them to behave as individual units. In contrast, intermolecular hydrogen bonding facilitates chain propagation, leading to higher surface coverage and the formation of a more cohesive protective film, thereby enhancing corrosion inhibition efficiency. A similar stabilizing effect of intermolecular hydrogen bonding on the integrity of protective layers has been reported by Yan and co-workers,^45^ who demonstrated that hydrogen-bonded networks contribute to the robustness of the adsorbed film and improve its barrier properties against corrosive species.

This behavior is consistent with the findings of Bilton et al.^46^ and Nagy,^47^ who showed that stable intramolecular hydrogen bonds, particularly those forming conjugated six-membered rings, reduce the availability of functional groups for intermolecular hydrogen bonding and external interactions. Consequently, the reduced ability of adsorbed molecules to establish an extended intermolecular hydrogen bonding network may lead to the formation of a less cohesive protective film. This interpretation is further supported by the calculated interatomic distances (Figure 6), which indicate the formation of stable intramolecular hydrogen bonds in the **4a–e** series, consistent with the lower inhibition efficiencies observed for these compounds compared with the **3a–d** series.

Interestingly, compound **4e** exhibited an inhibition efficiency of only 41%, despite being the softest compound in the series and exhibiting the lowest Δ*E*, even when compared with compounds **3a–d** (Table 2). Although its calculated physicochemical parameters suggest superior performance relative to compounds **4a–d**, its inhibition efficiency remains considerably lower than that of compounds **3a–d**. The optimized structure of compound **4e** reveals not only the previously discussed intramolecular hydrogen bond but also an additional interaction between the sulfonyl oxygen atoms and the amino hydrogen (SO_2_···H–N) (Figure 5), further supporting the hypothesis that intramolecular hydrogen bonding plays an important role in determining the corrosion inhibition efficiency of these compounds.

An additional observation supporting this hypothesis is that, in compound **3a**, the distance between the sulfur atom and the hydrogen atom of the amino group (N–H···S) is 2.285 Å, a value consistent with the formation of an intramolecular hydrogen bond.^48^ This interaction may also contribute to the lower inhibition efficiency of compound **3a** relative to the other members of the **3a–d** series.

Overall, the quantum chemical descriptors are in good agreement with the experimental inhibition efficiencies, indicating that the corrosion performance of the synthesized compounds is governed not only by their global electronic properties but also by the distribution of electron density and specific structural features, such as intramolecular hydrogen bonding. Similar correlations between DFT descriptors and experimental corrosion inhibition behavior have been observed in recent combined experimental–DFT studies of organic inhibitors.^37–39,41,42^

The surface coverage (θ) was used to investigate the adsorption behavior of the inhibitor on the metal surface and to identify the adsorption isotherm that best describes the process. The experimental data were fitted to the Langmuir, Freundlich, and Flory–Huggins isotherm models (eqs 8–10), and the corresponding plots are presented in Figure 7.

**Figure 7:**
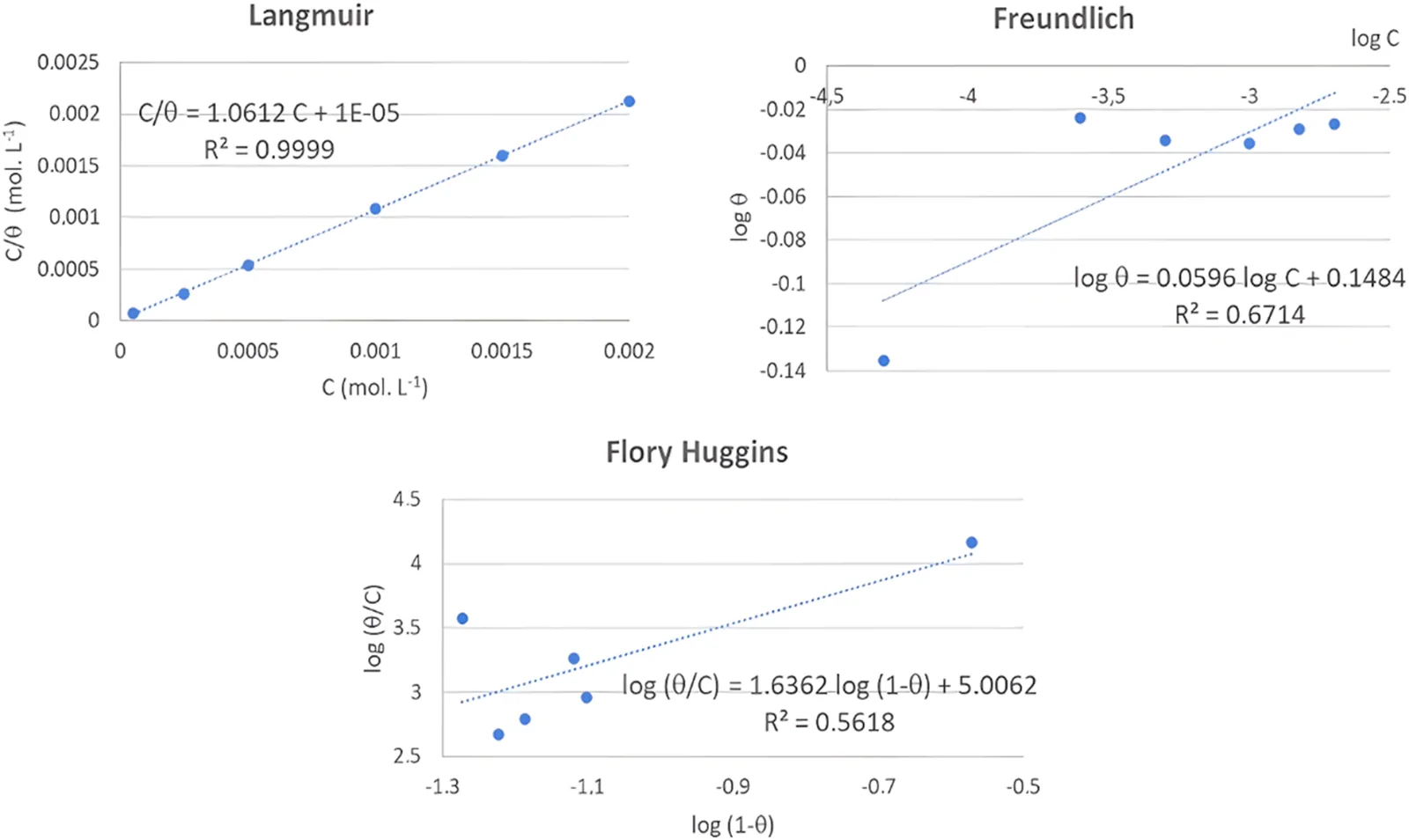
Adsorption data of inhibitor **3d** on SAE 1020 steel at 25 °C fitted to the Langmuir, Freundlich and Flory–Huggins adsorption isotherm models.

Among the evaluated models, the Langmuir adsorption isotherm provided the best fit to the experimental data, with a correlation coefficient (*R* ^2^) of 0.9999. This excellent agreement indicates that adsorption of compound **3d** occurs predominantly through the formation of a uniform monolayer on the SAE 1020 steel surface.^49^

The standard Gibbs free energy of adsorption (Δ*G*°_ads_) was calculated using eq 11. This thermodynamic parameter provides valuable information about the adsorption mechanism of the inhibitor on the metal surface. According to the literature, Δ*G*°_ads_ values higher than −20 kJ mol^–1^ are characteristic of physisorption, values between −20 and −40 kJ mol^–1^ indicate the coexistence of physisorption and chemisorption, and values lower than or equal to −40 kJ mol^–1^ are indicative of chemisorption.^33^

The calculated Δ*G*°_ads_ value for inhibitor **3d**, obtained from the *K*_ads_ derived from the Langmuir adsorption isotherm, was −39.027 kJ mol^–1^. The negative value confirms the spontaneous nature of the adsorption process, whereas its magnitude indicates that the interaction between inhibitor **3d** and the SAE 1020 steel surface involves a combination of physisorption and chemisorption. These findings are consistent with those reported by Aslam and co-workers,^17^ who, in their review of pyrazole derivatives as corrosion inhibitors, showed that most compounds in this class follow the Langmuir adsorption model and adsorb through either chemisorption or a mixed adsorption mechanism involving both physical and chemical interactions.

## 4 Conclusions

Nine polysubstituted pyrazoles (**3a**–**d** and **4a**–**e**) were synthesized in 43–91% yields through double vinylic substitution of ketene dithioacetals followed by cyclization. Compounds **3a**–**d** exhibited corrosion inhibition efficiencies higher than 75% in gravimetric tests performed in 1.0 mol L^–1^ HCl at an inhibitor concentration of 2.0 mmol L^–1^.

The results demonstrated that the substituent at the R2 position plays a key role in determining the corrosion inhibition efficiency of these polysubstituted pyrazoles. Compounds belonging to the **4a**–**e** series, in which the carbonyl group forms an intramolecular hydrogen bond with the amino group at the R4 position, exhibited significantly lower inhibition efficiencies. This behavior is attributed to the reduced ability of the adsorbed molecules to establish an extended intermolecular hydrogen bonding network, resulting in the formation of a less cohesive protective film.

Compound **3d** exhibited the highest inhibition efficiency at 2.0 mmol L^–1^ and maintained inhibition efficiencies above 90% even at a concentration as low as 0.25 mmol L^–1^. Potentiodynamic polarization measurements showed that **3d** behaves predominantly as a cathodic inhibitor at low concentrations and as a mixed-type inhibitor up to 0,50 mmol L^–1^. In addition, SEM micrographs revealed a substantially lower amount of corrosion products on the steel surface in the presence of **3d**, corroborating the gravimetric and electrochemical results.

The adsorption behavior of compound **3d** was well described by the Langmuir adsorption isotherm, and the calculated Δ*G*°_ads_ value indicated that adsorption occurs through a mixed mechanism involving both physisorption and chemisorption.

Overall, these findings demonstrate that pyrazole derivatives synthesized from ketene dithioacetals constitute promising molecular platforms for the development of efficient corrosion inhibitors for carbon steel in acidic media.

## Supplementary Material



## Data Availability

The data supporting the findings of this study are available in the Supporting Information associated with this article.

## Notes

Infrared, Mass, ^1^H NMR and ^13^C NMR spectra related to compound **3a**–**d** and **4a**–**e** (Figures S1–S24) (PDF)
