# Supplementary material for: Polysubstituted Pyrazoles as Corrosion Inhibitors for SAE 1020 Steel in 1 M HCl
Source: ACS Omega. 2026 Aug 22;11(35):53477–86. doi: 10.1021/acsomega.6c06392 (PMC13563934; doi:10.1021/acsomega.6c06392)

Supporting Information

Polysubstituted pyrazoles as corrosion inhibitors for SAE 1020 steel in 1 M HCl

Gabriel R. Antunes,[a] Ana J. F. Souza,[a] Larissa L. Machado,[a] Fillipe A. Carmo,[a] Alfredo G. M. Castro,[b] Denise C. Bertagnolli,[a] Elivelton A. Ferreira,[a,b] Julliane Yoneda,[a] Diego Pereira Sangi [a]*

[a] Departamento de Química, Instituto de Ciências Exatas, Universidade Federal Fluminense, 27213-145, Volta Redonda, Rio de Janeiro, Brazil.
[b] Programa de Pós-graduação em Engenharia Metalúrgica, Universidade Federal Fluminense, 27255-125, Volta Redonda, Rio de Janeiro, Brazil.

* Corresponding author: dpsangi@id.uff.br ; tel (24) 981257650 https://orcid.org/0000-0003-0388-5409.

Figure S1. ^{1}H NMR (400 MHz) spectrum of 5-amino-4-cyano-3-(methylthio)-1H-pyrazole-1-carbothioamide in DMSO-d_6 (**3a**).

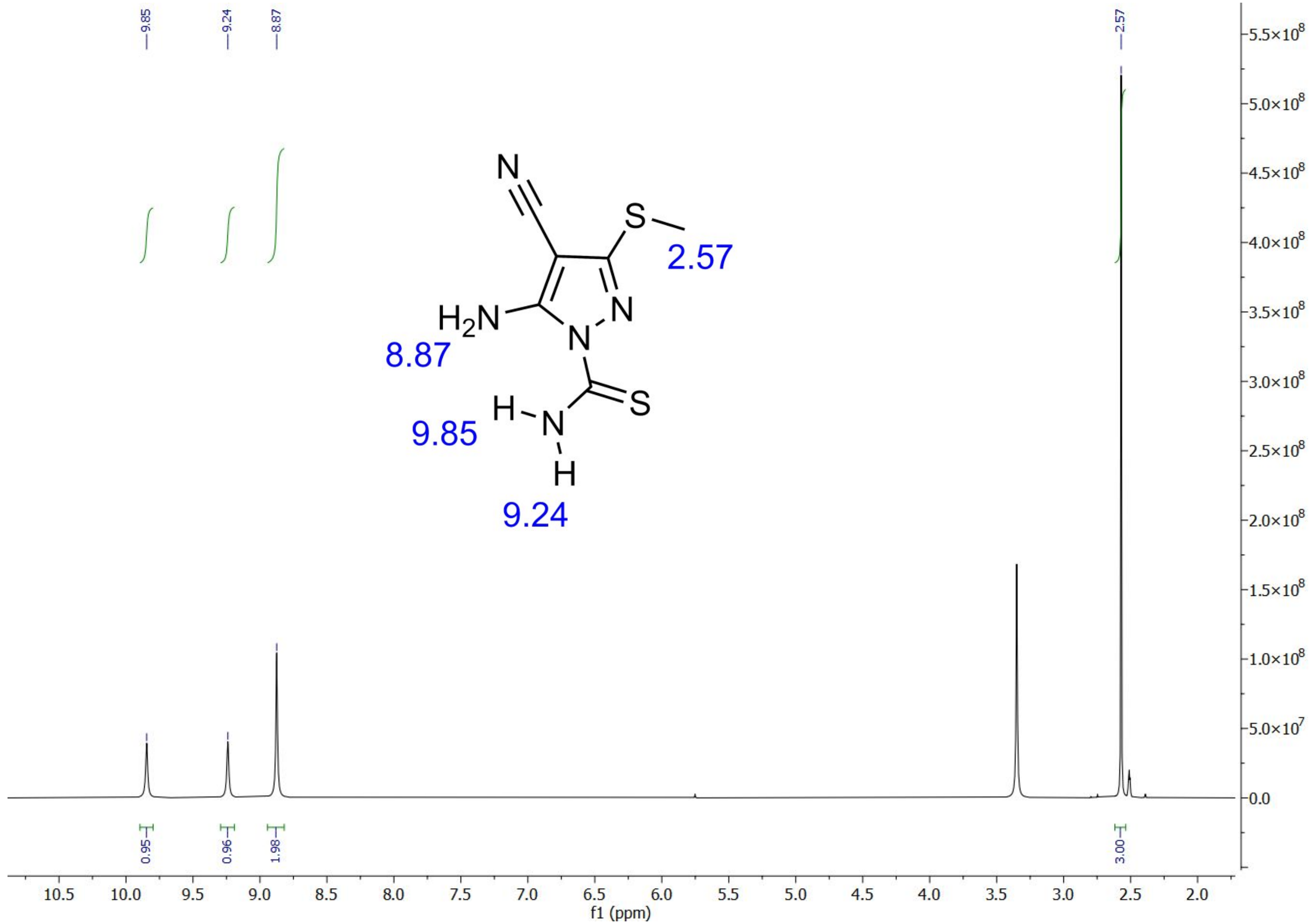

Figure S2. ^{13}C NMR (101 MHz) spectrum of 5-amino-4-cyano-3-(methylthio)-1H-pyrazole-1-carbothioamide in DMSO-d_6 (**3a**).

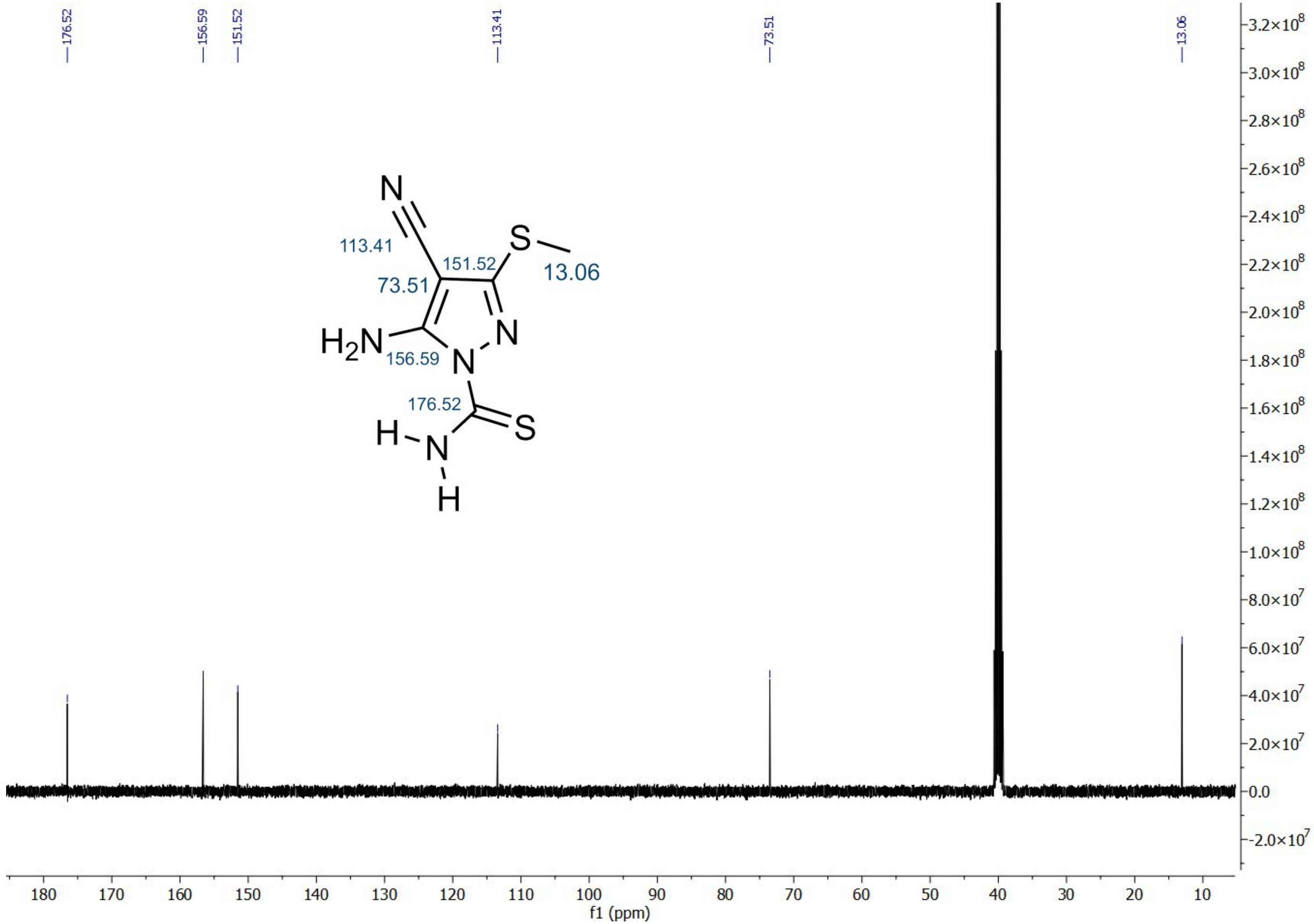

Figure S3. ^{1}H NMR (400 MHz) spectrum of 5-amino-3-(methylthio)-1-phenyl-1H-pyrazole-4-carbonitrile in $CDCl_3$ (**3b**).

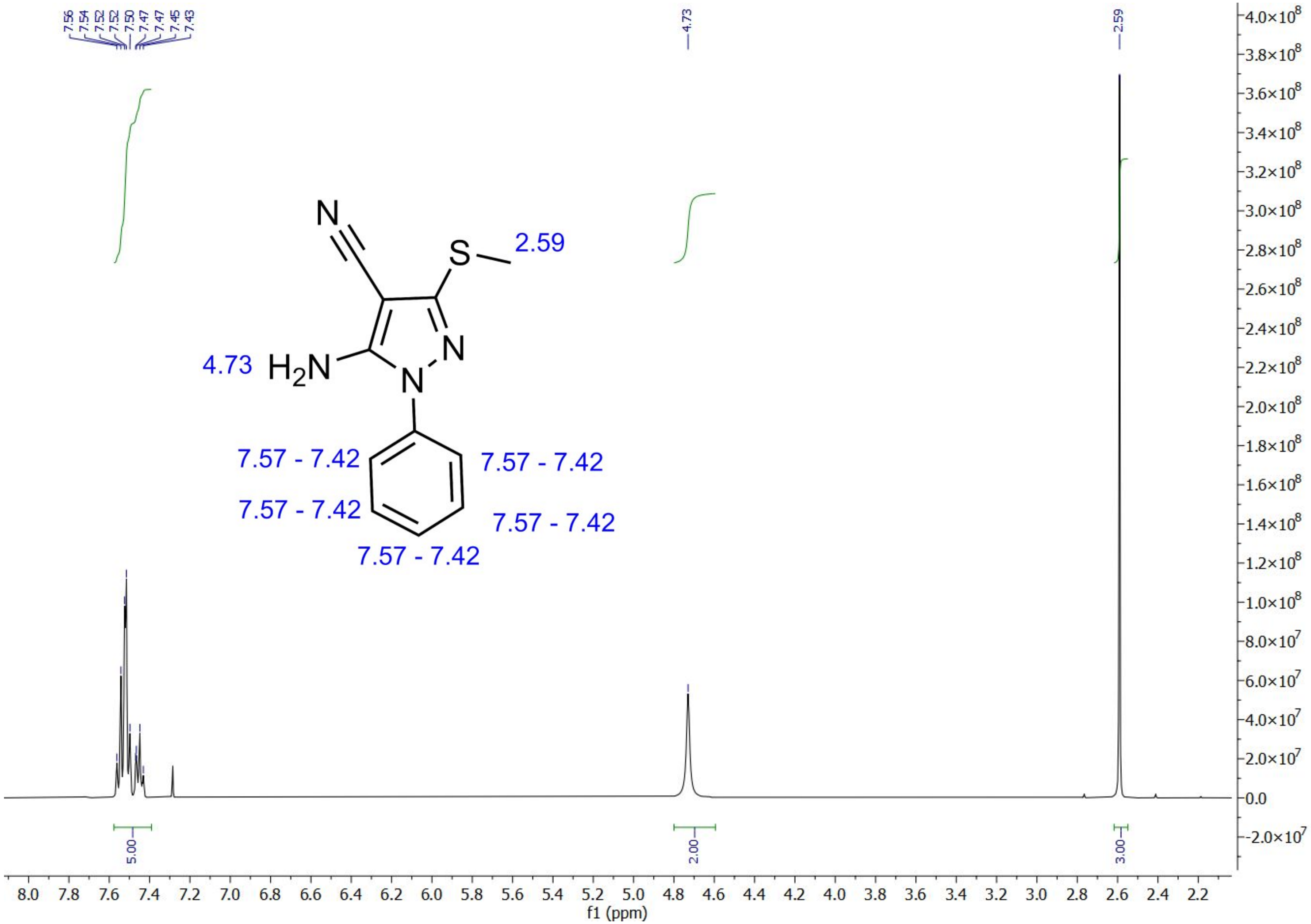

Figure S4. ^{13}C NMR (101 MHz) spectrum of methyl 5-amino-3-(methylthio)-1-phenyl-1H-pyrazole-4-carboxylate in $CDCl_3$ (**3b**).

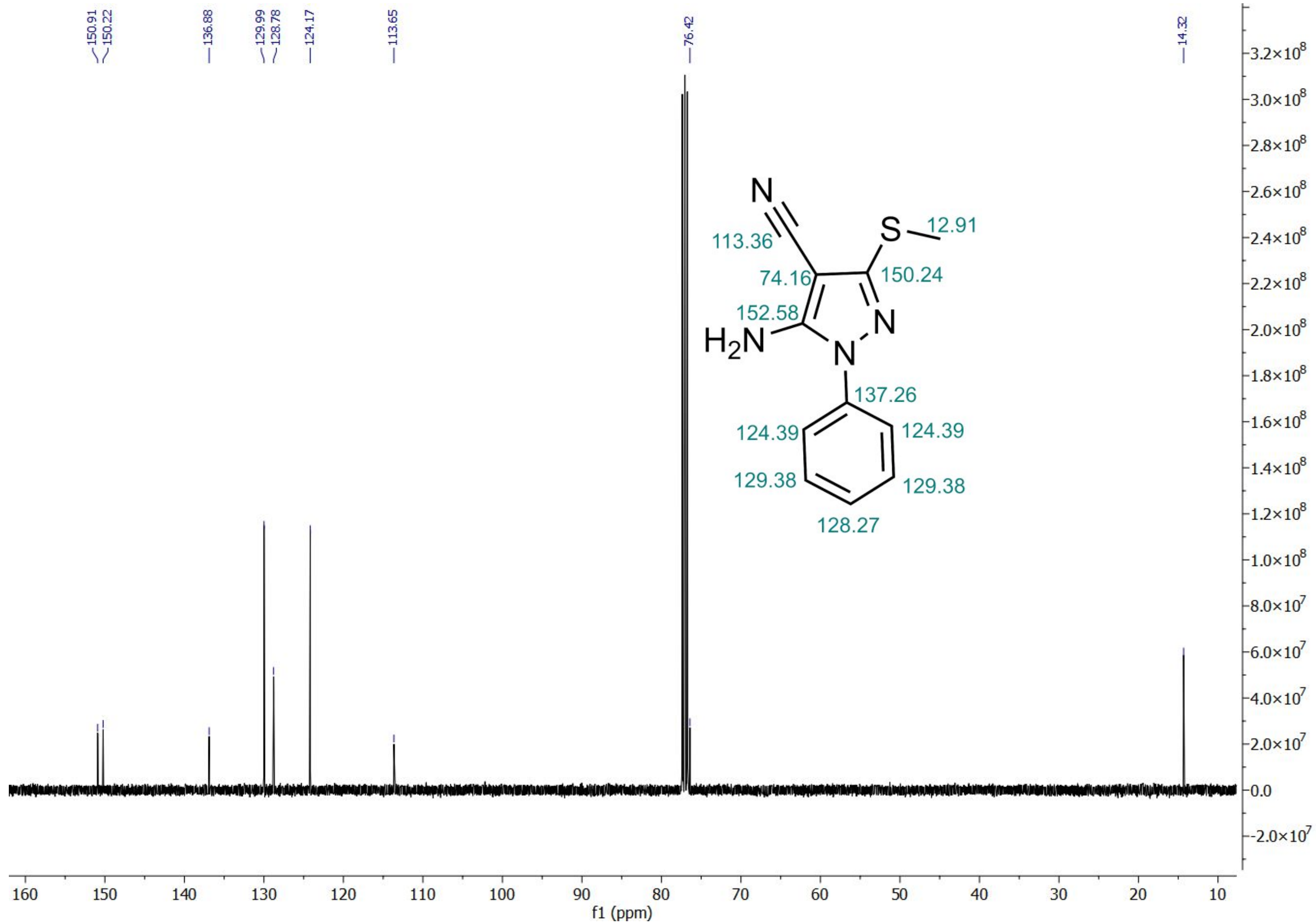

Figure S5. ^{1}H NMR (400 MHz) spectrum of 5-amino-3-(methylthio)-1H-pyrazole-4-carbonitrile in DMSO

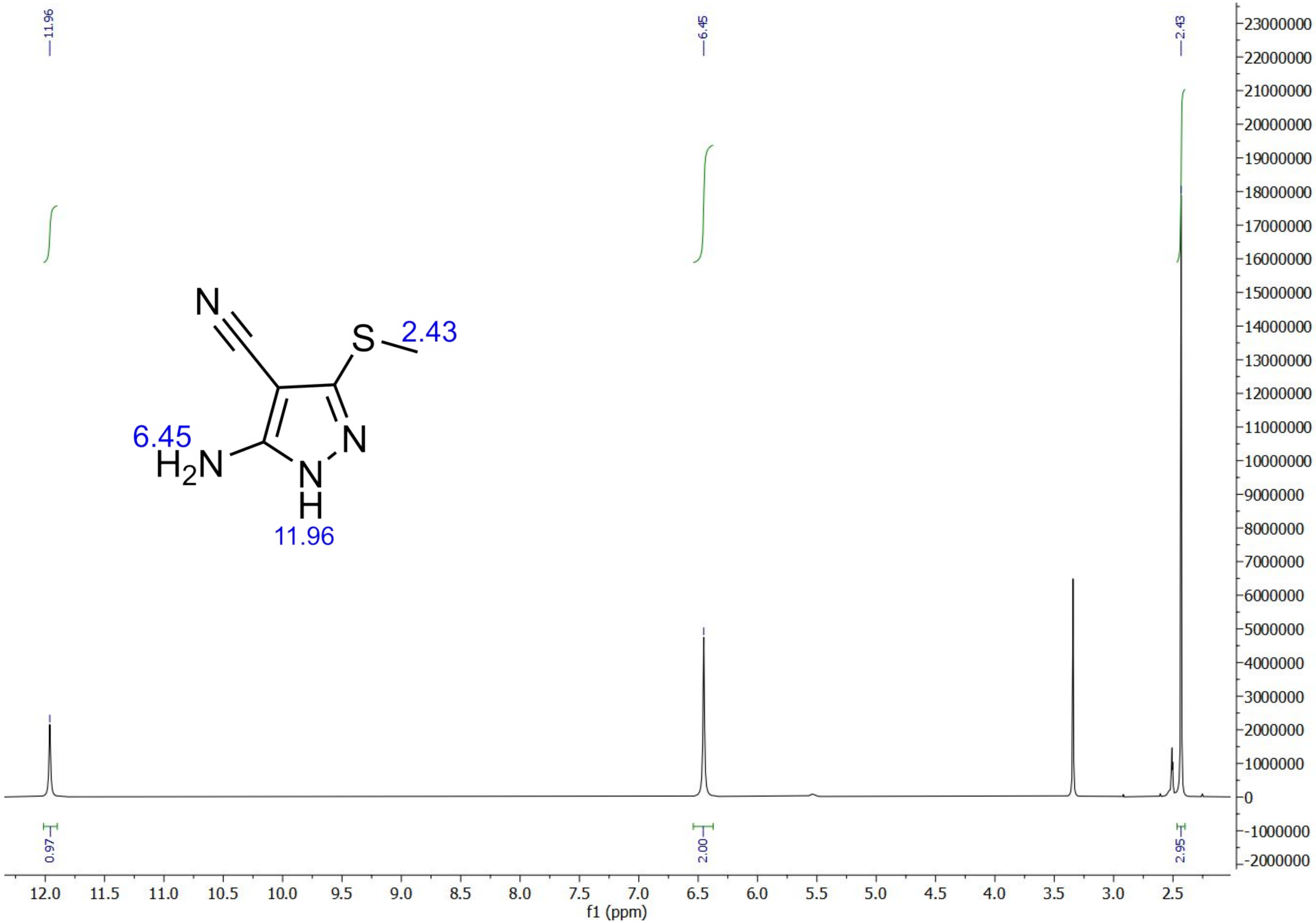

Figure S6. ^{13}C NMR (101 MHz) spectrum of 5-amino-3-(methylthio)-1H-pyrazole-4-carbonitrile in DMSO-d_6 (**3c**).

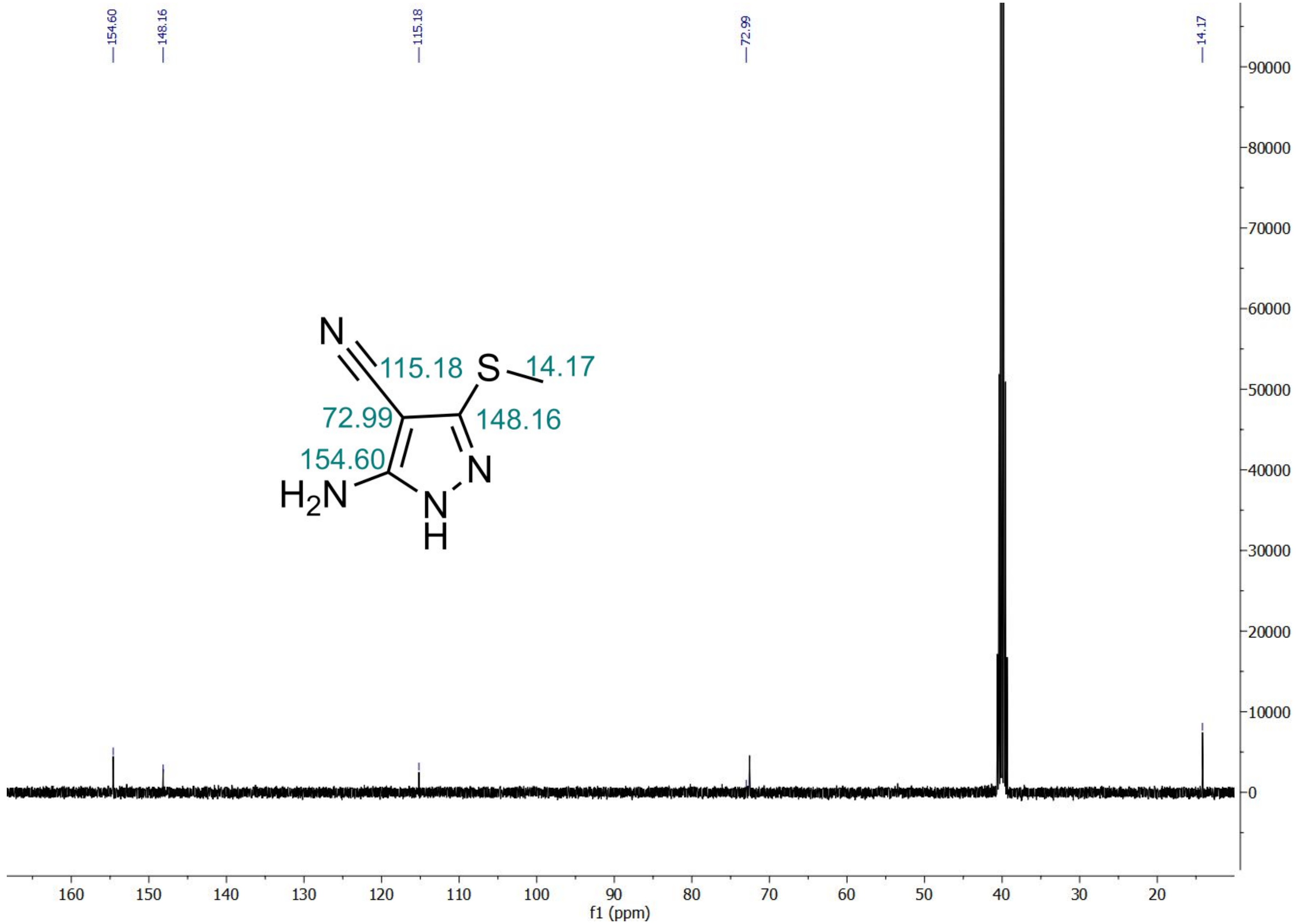

Figure S7. ^{1}H NMR (400 MHz) spectrum of 3-(methylthio)-5-phenyl-1H-pyrazole-4-carbonitrile in DMSO- d_6 (**3d**).

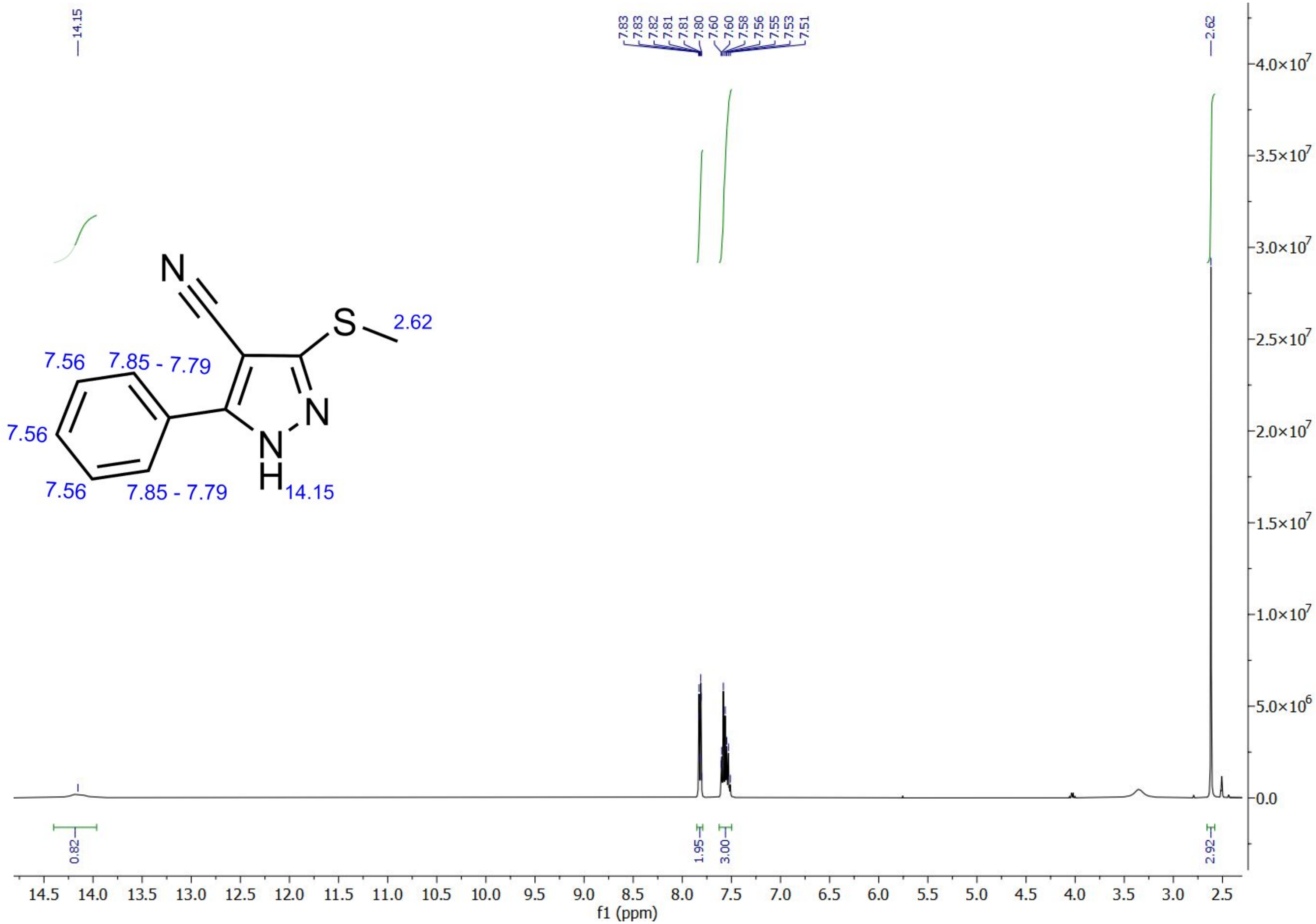

Figure S8. ^{1}H NMR (400 MHz) spectrum of 3-(methylthio)-5-phenyl-1H-pyrazole-4-carbonitrile in DMSO- d_6 expansion (**3d**).

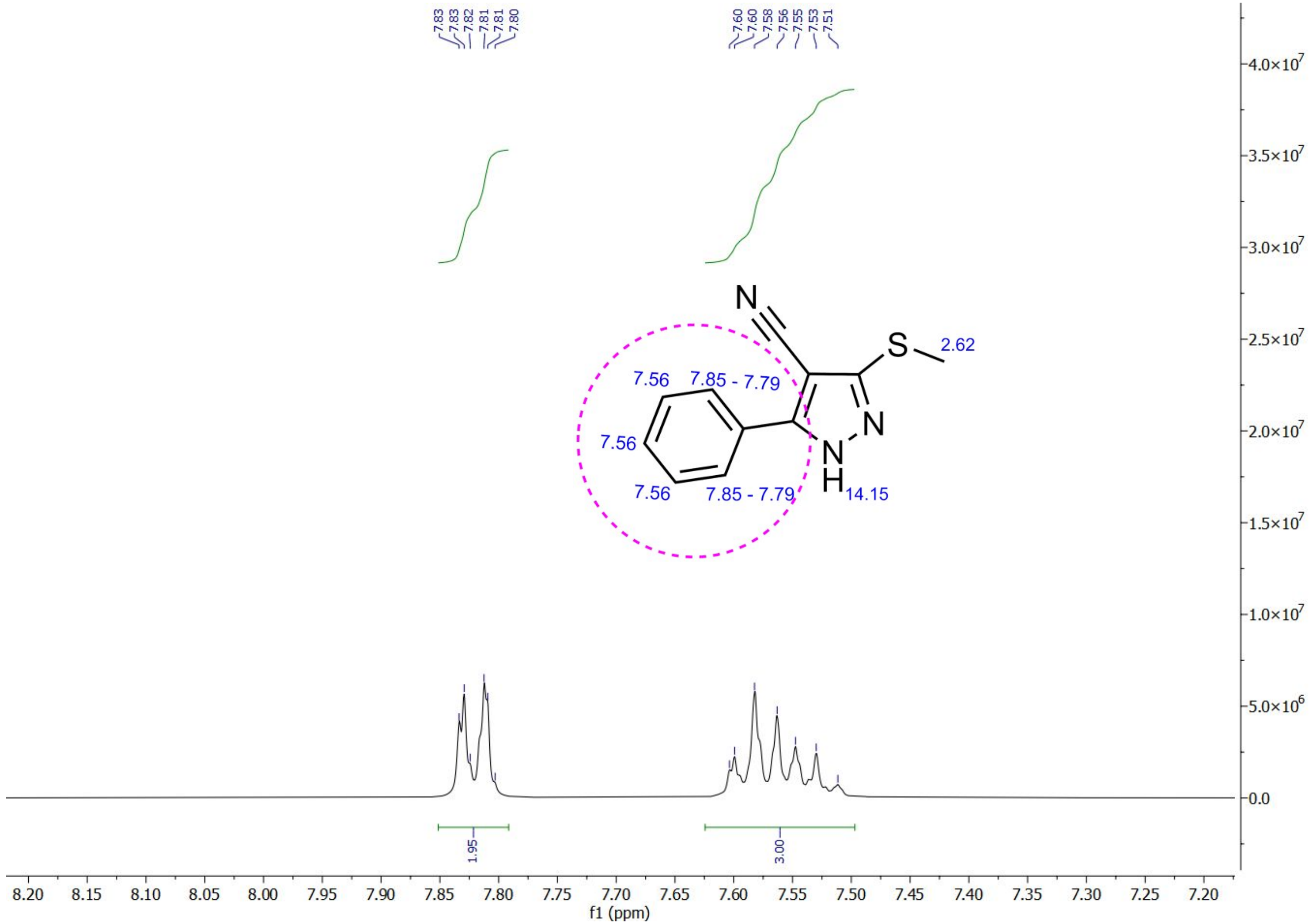

Figure S9. ^{13}C NMR (101 MHz) spectrum of 3-(methylthio)-5-phenyl-1H-pyrazole-4-carbonitrile in DMSO- d_6 (**3d**).

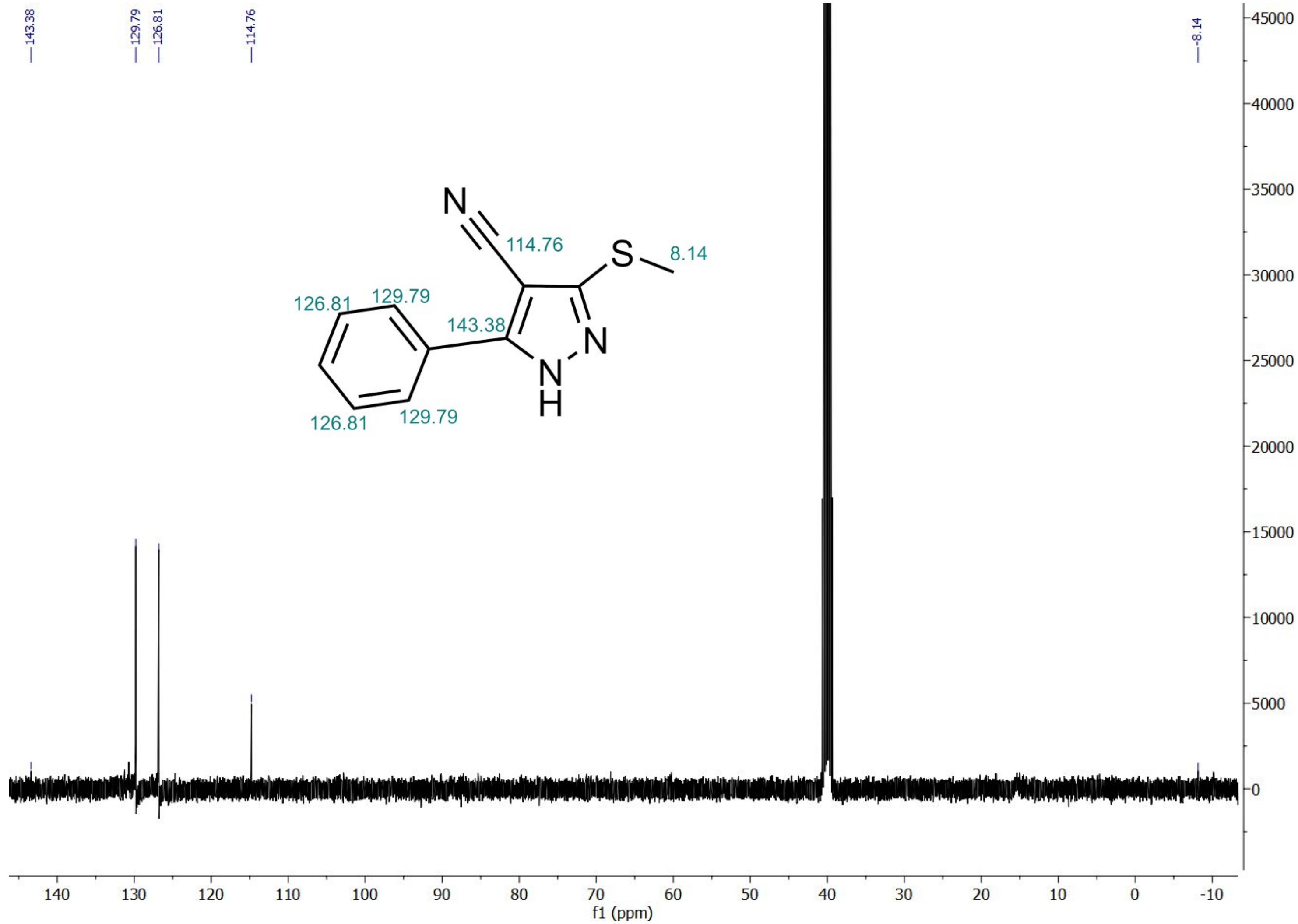

Figure S10. Infrared spectrum of of 3-(methylthio)-5-phenyl-1H-pyrazole-4-carbonitrile (**3d**).

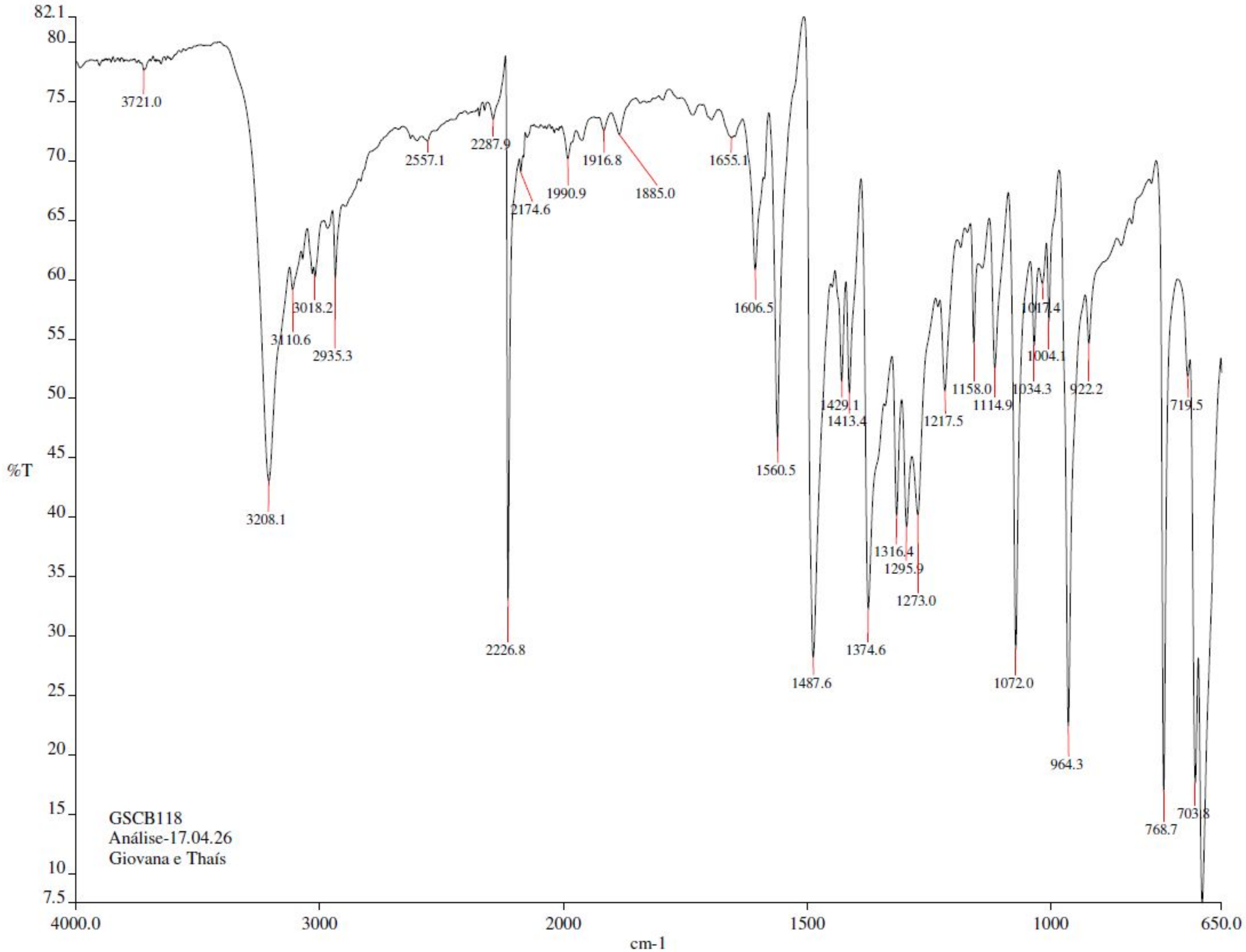

Figure S11. High resolution mass spectrum of of 3-(methylthio)-5-phenyl-1H-pyrazole-4-carbonitrile (**3d**).

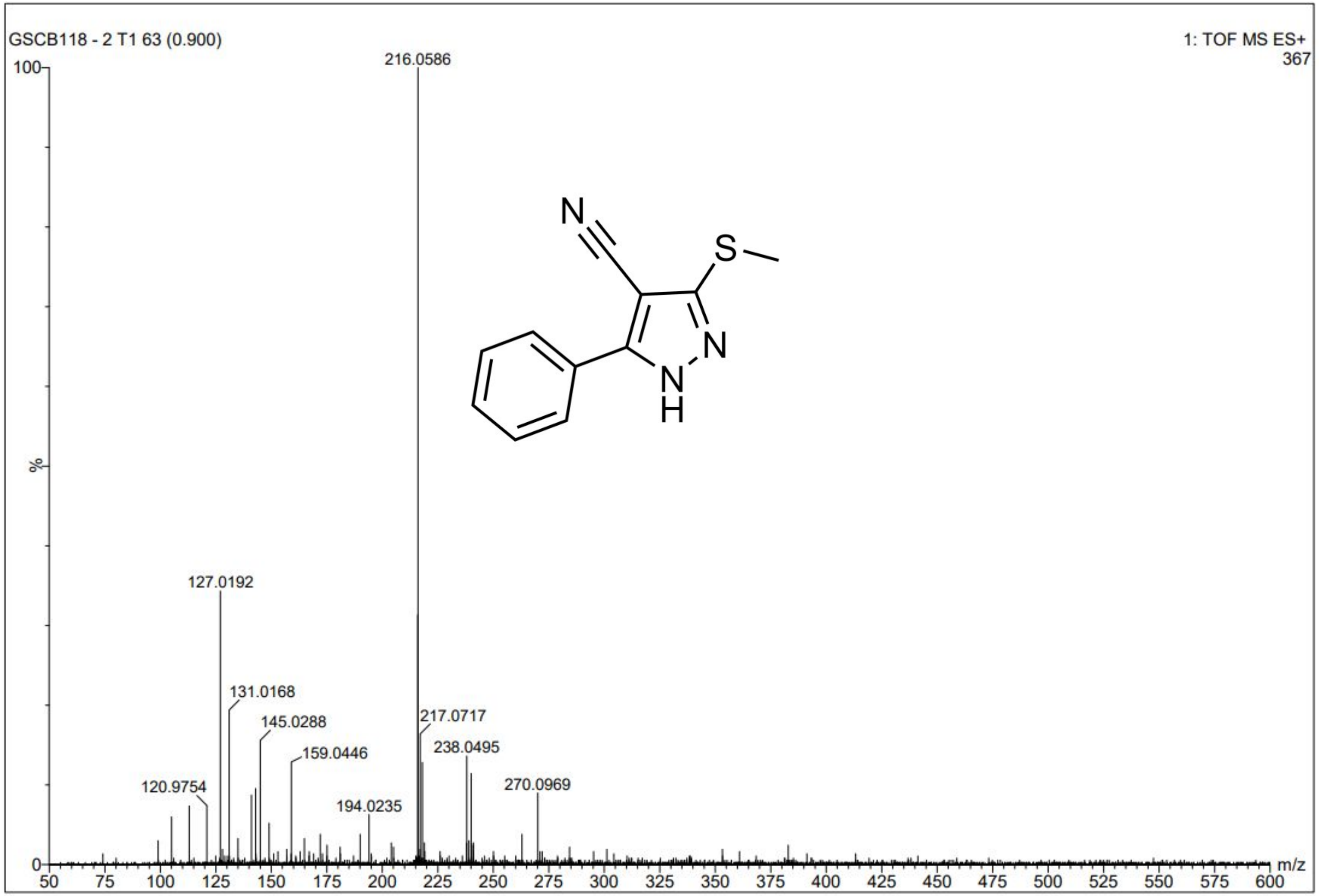

Figura S12. ^{1}H NMR (400 MHz) spectrum of methyl 5-amino-3-(methylthio)-1H-pyrazole-4-carboxylate in DMSO-d_6 (**4a**)

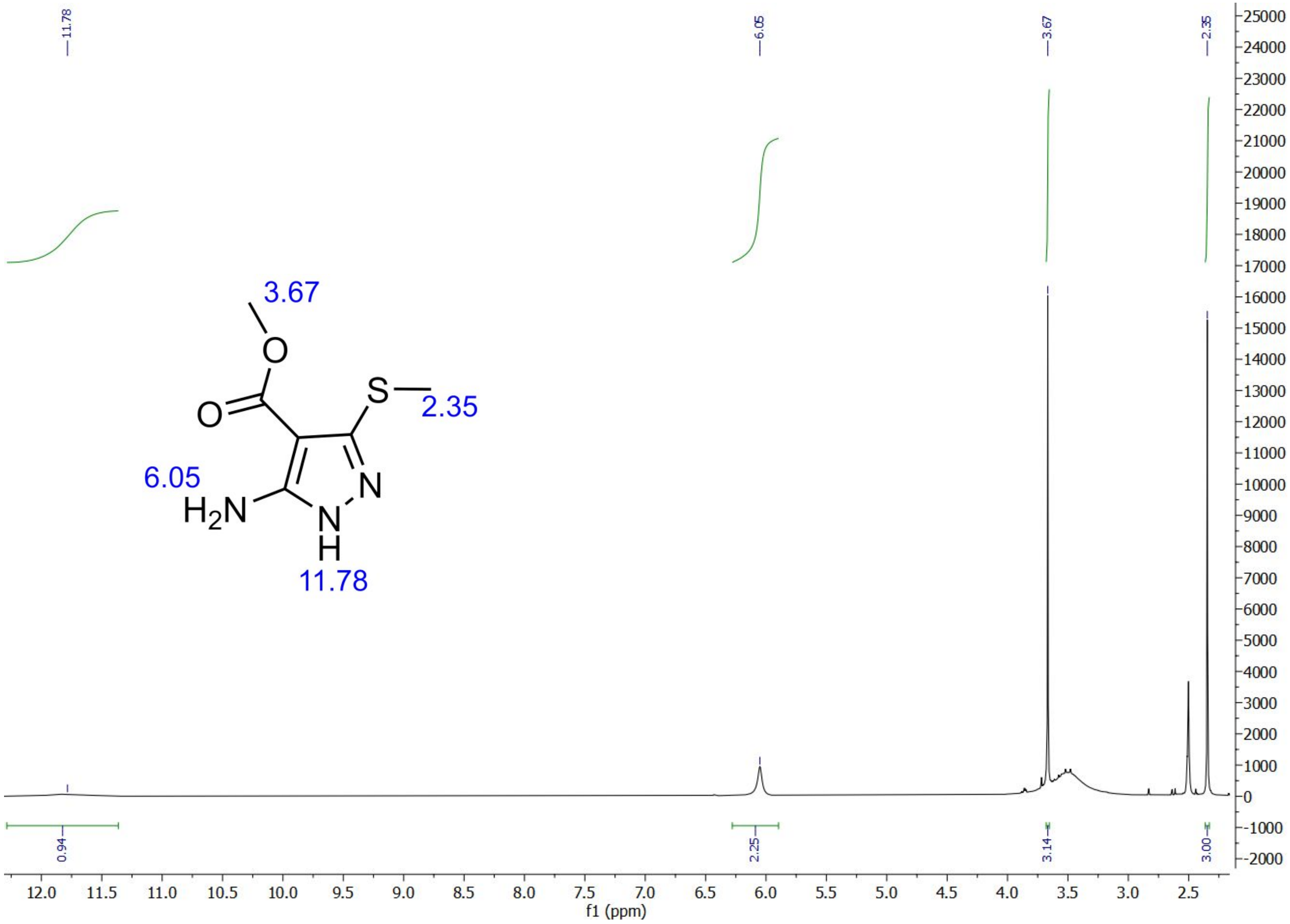

Figura S13. ^{13}C NMR (101 MHz) spectrum of methyl 5-amino-3-(methylthio)-1H-pyrazole-4-carboxylate in DMSO-d_6 (**4a**).

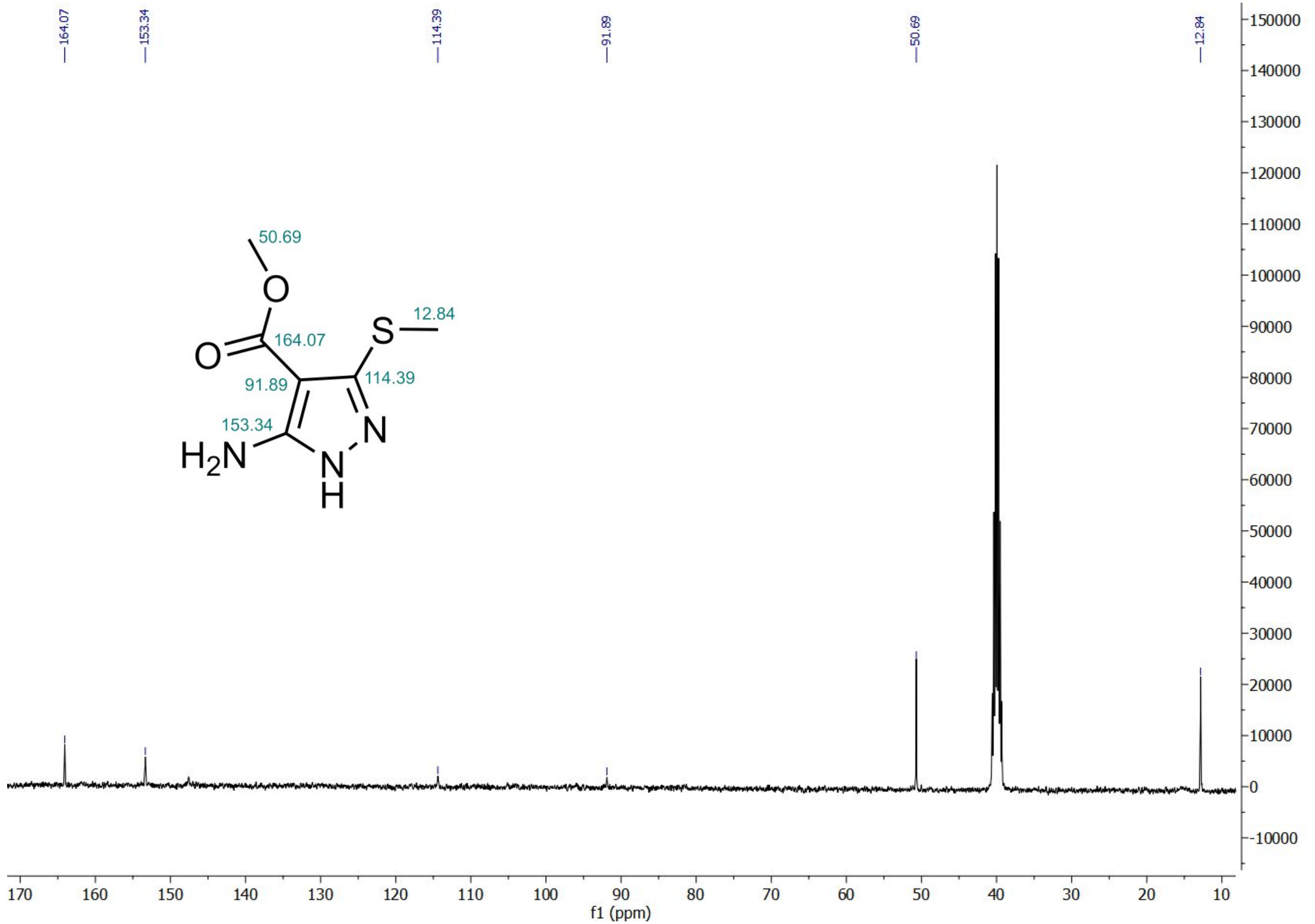

Figura S14. ^{1}H NMR (400 MHz) spectrum of (5-amino-3-(methylthio)-1-phenyl-1H-pyrazol-4-yl)(phenyl)methanone in DMSO-d_6 (**4b**).

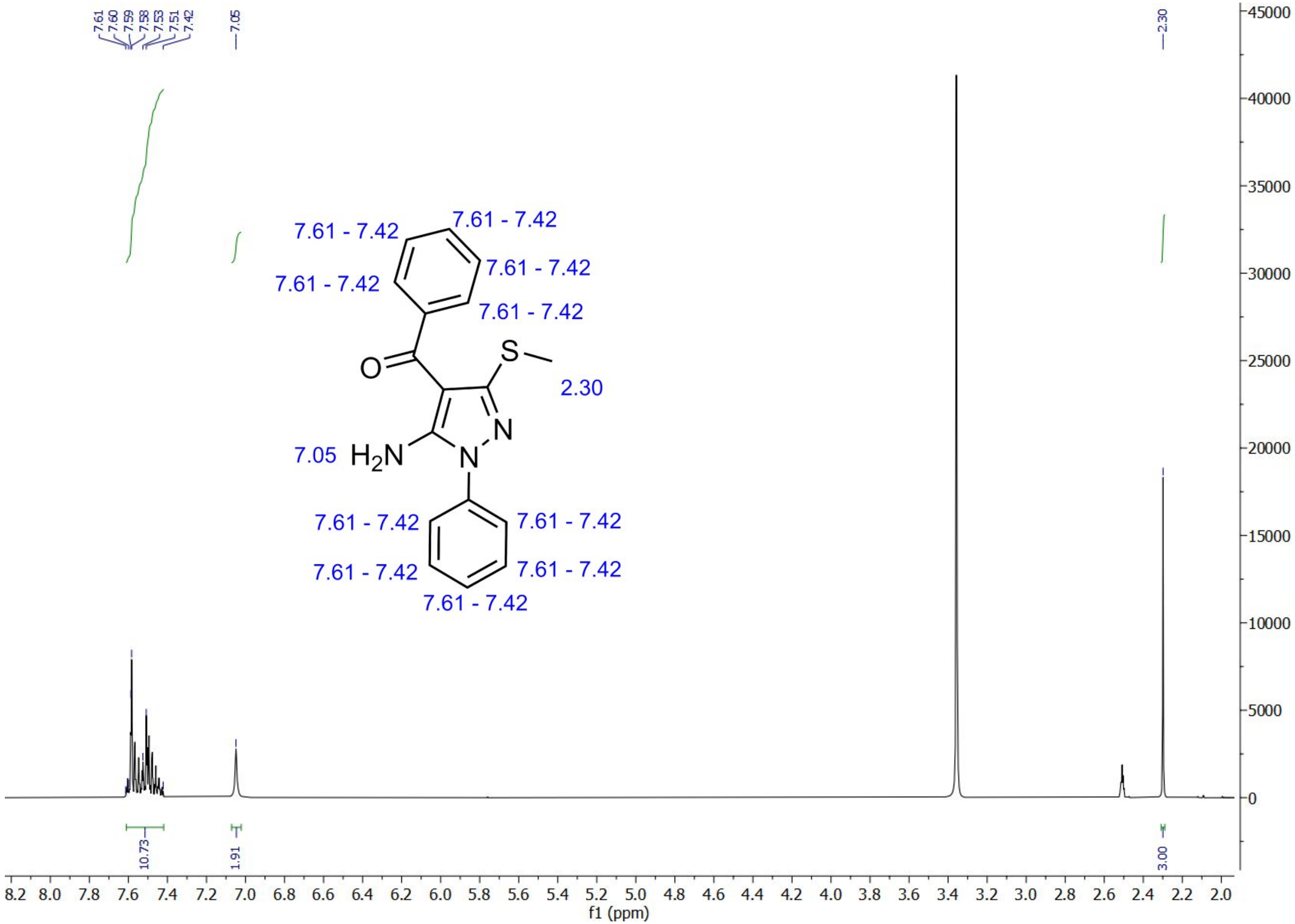

Figura S15. ^{13}C NMR (101 MHz) spectrum of (5-amino-3-(methylthio)-1-phenyl-1H-pyrazol-4-yl)(phenyl)methanone in DMSO- d_6 (**4b**).

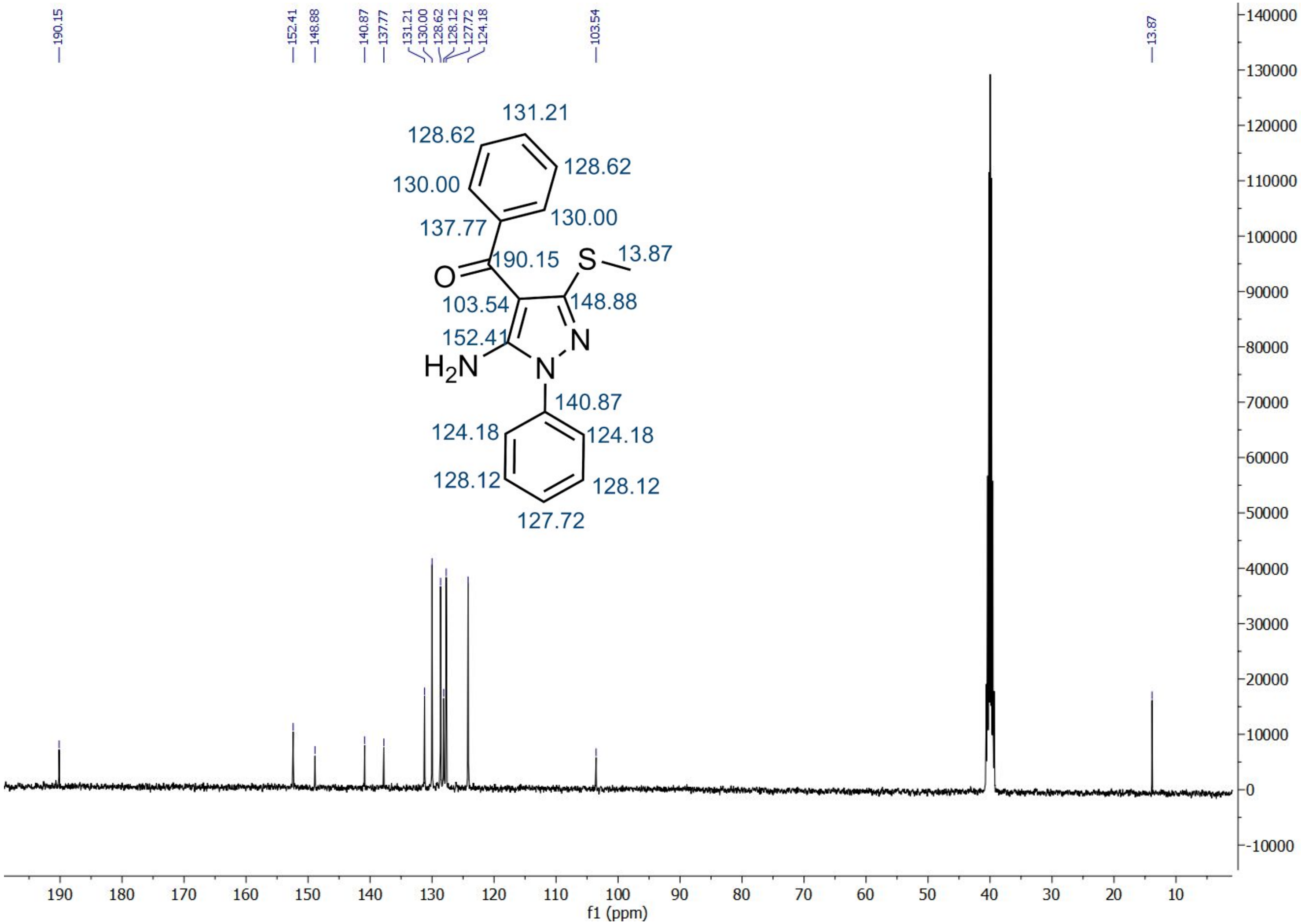

Figura S16. ^{1}H NMR (400 MHz) spectrum of methyl 5-amino-3-(methylthio)-1-phenyl-1H-pyrazole-4-carboxylate in DMSO- d_6 (**4c**).

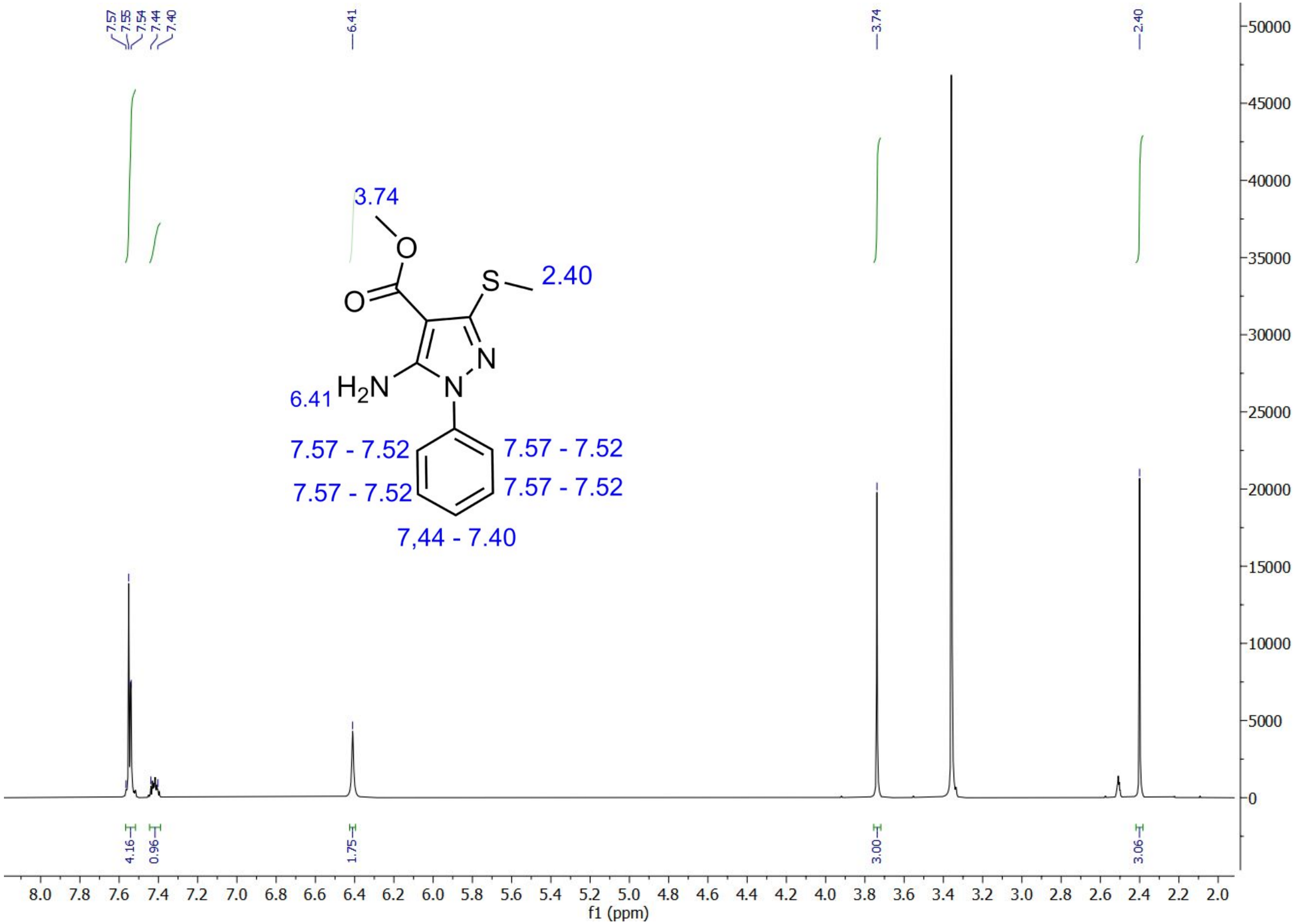

Figura S17. ^{13}C NMR (101 MHz) spectrum of methyl 5-amino-3-(methylthio)-1-phenyl-1H-pyrazole-4-carboxylate in $CDCl_3$ (**4c**).

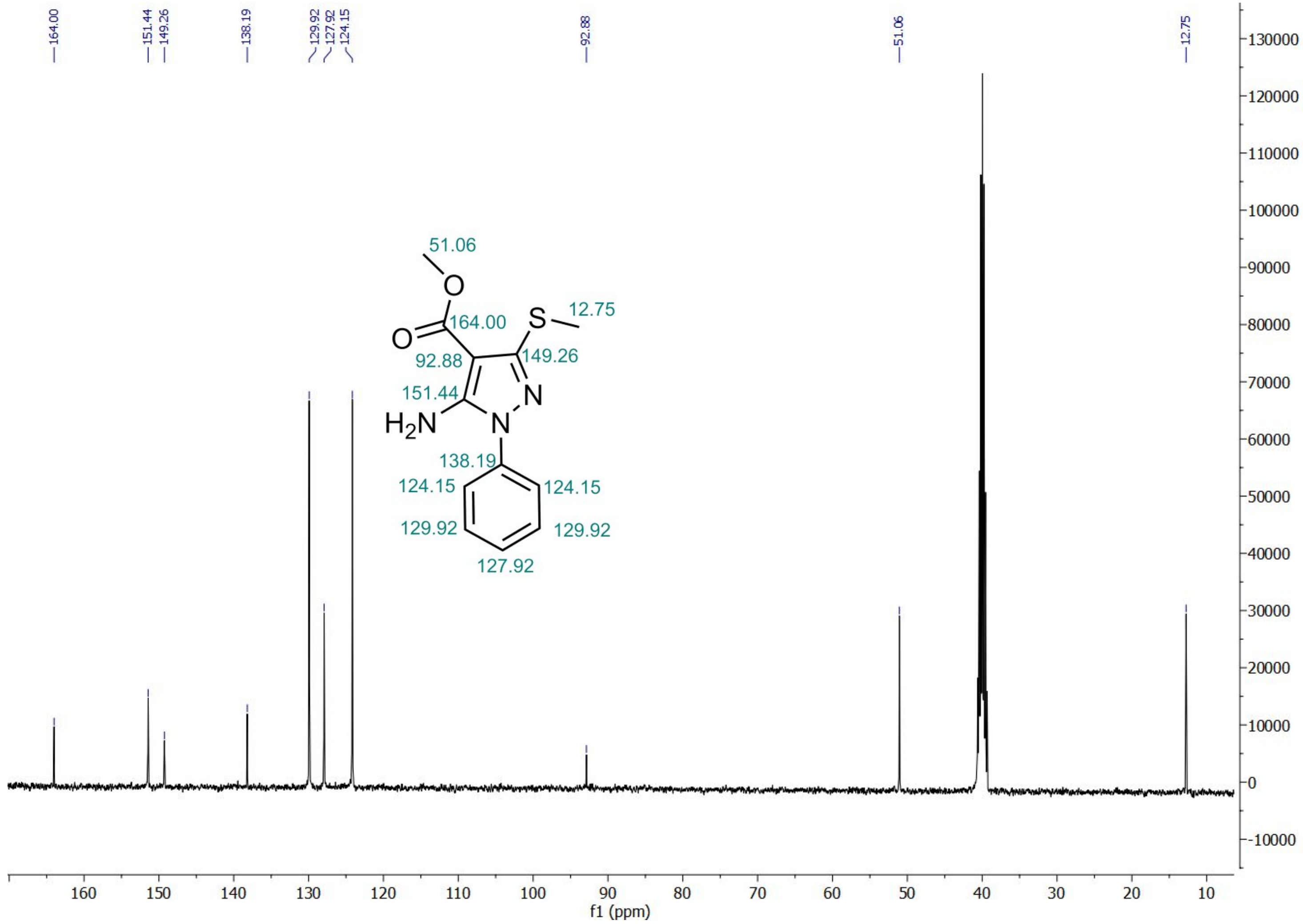

164.00
151.44
149.26
138.19
129.92
127.92
124.15
92.88
51.06
12.75
51.06
O
12.75
S
O
164.00
92.88
149.26
151.44
N
H2N
N
138.19
124.15
124.15
129.92
129.92
127.92
130000
120000
110000
100000
90000
80000
70000
60000
50000
40000
30000
20000
10000
0
-10000
160
150
140
130
120
110
100
90
80
70
60
50
40
30
20
10
f1 (ppm)

Figura S18. ^{1}H NMR (400 MHz) spectrum of methyl 5-amino-3-(methylthio)-1-(4-nitrophenyl)-1H-pyrazole-4-carboxylate in $CDCl_3$ (**4d**).

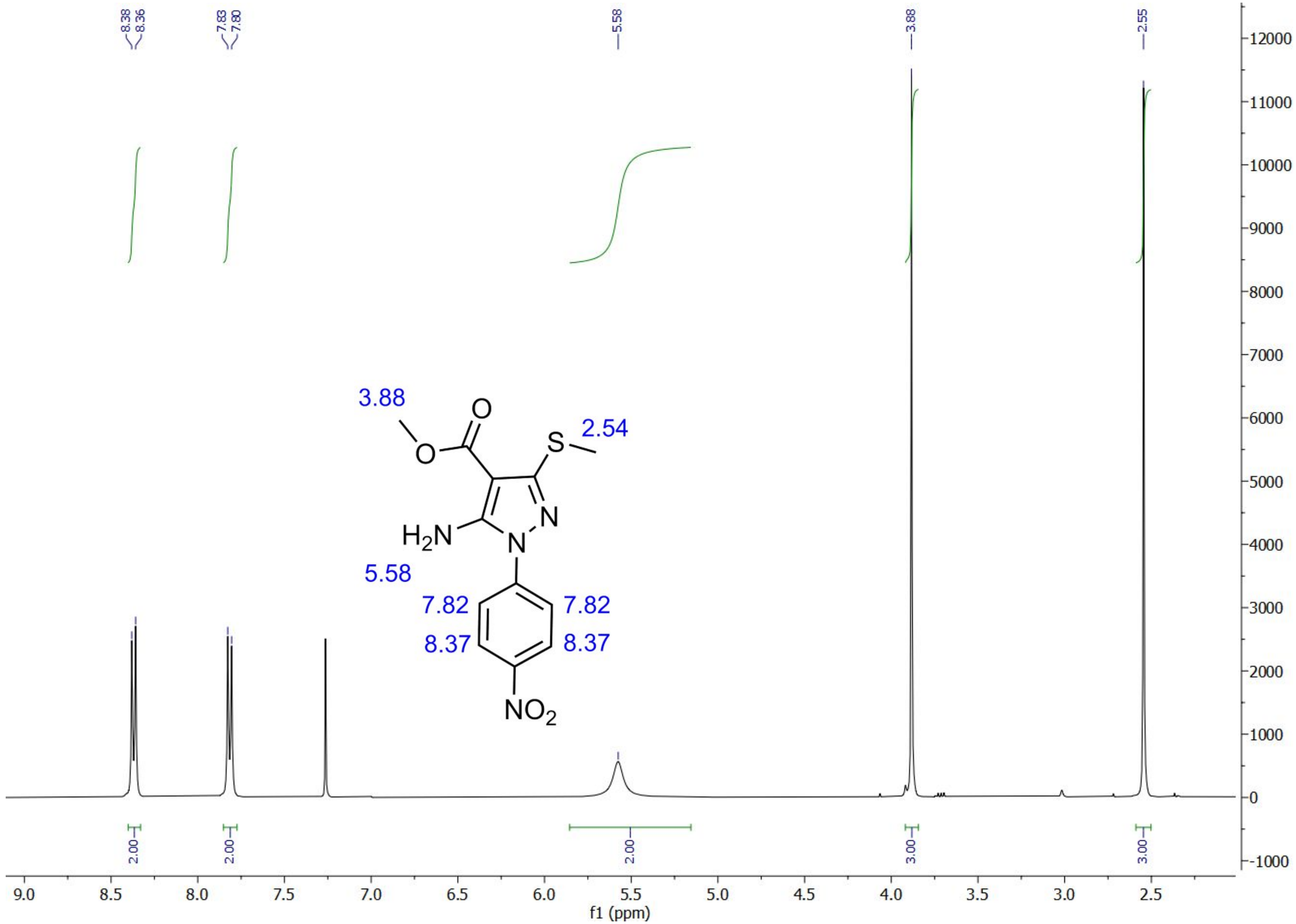

Figura S19. ^{13}C NMR (101 MHz) spectrum of methyl 5-amino-3-(methylthio)-1-(4-nitrophenyl)-1H-pyrazole-4-carboxylate in $CDCl_3$ (**4d**).

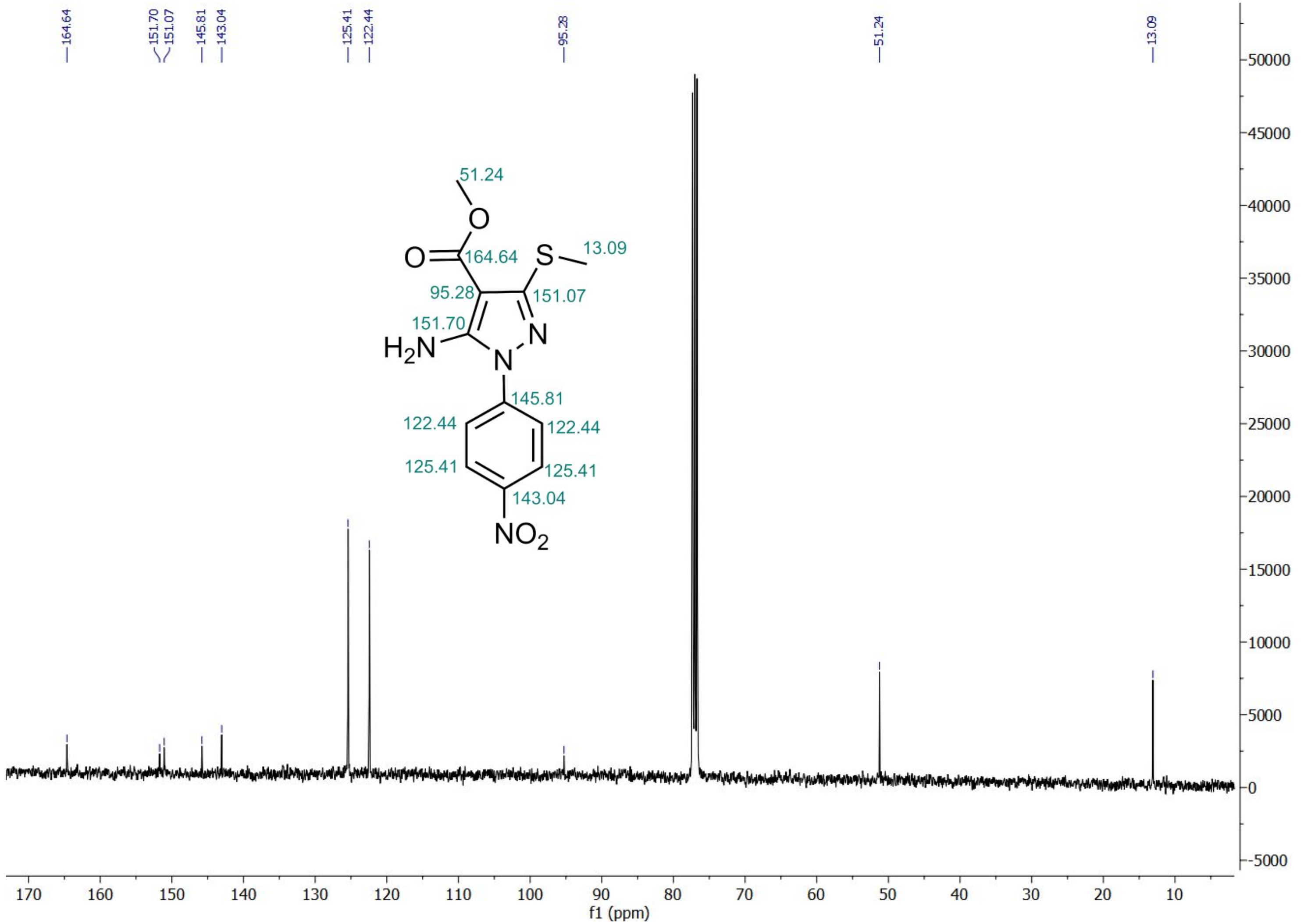

Figura S20. ^{1}H NMR (400 MHz) spectrum of (5-amino-3-(methylthio)-1-tosyl-1H-pyrazol-4-yl)(phenyl)methanone in $CDCl_3$ (**4e**).

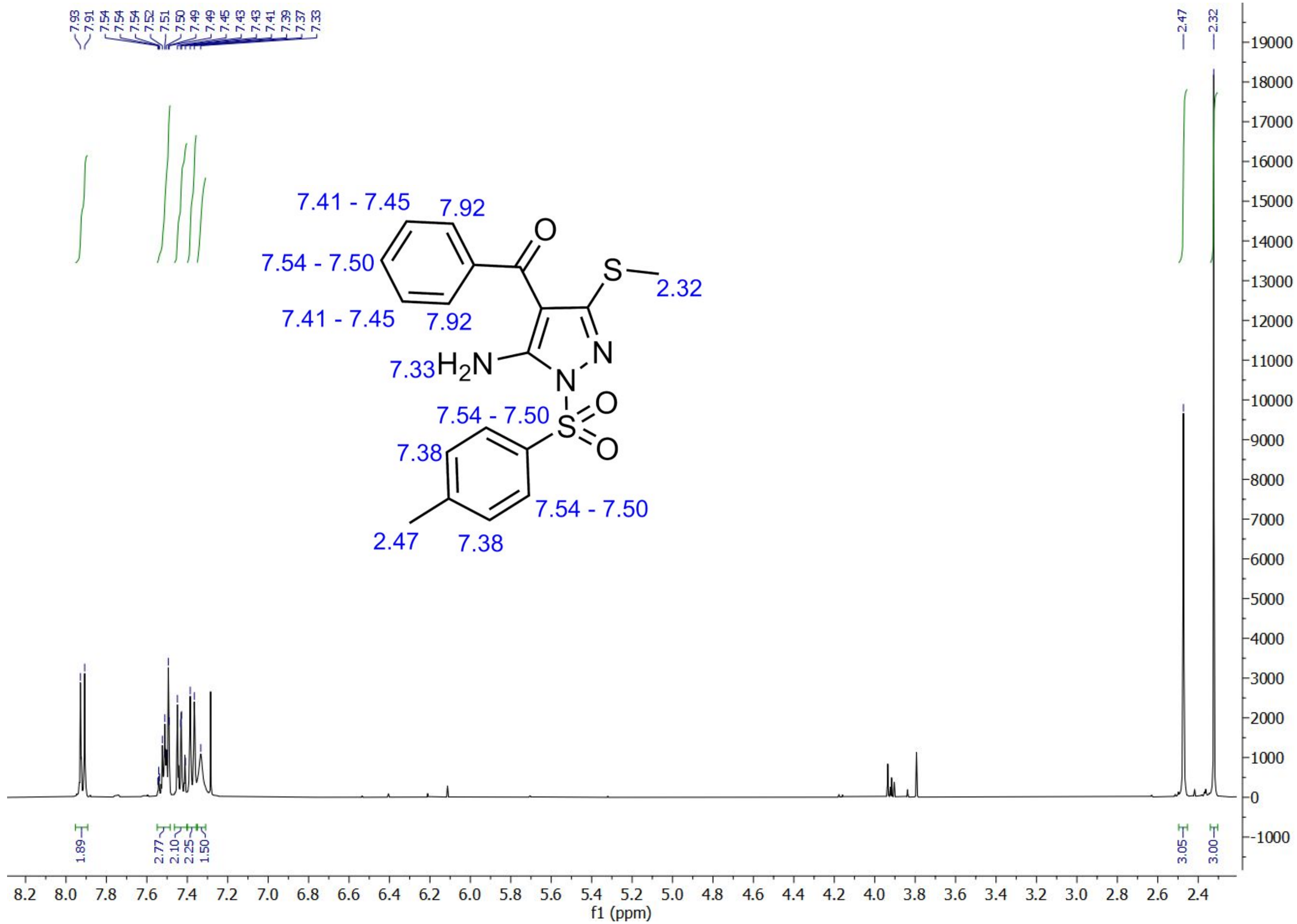

Figura S21. ^{1}H NMR (400 MHz) spectrum of (5-amino-3-(methylthio)-1-tosyl-1H-pyrazol-4-yl)(phenyl)methanone in $CDCl_3$ expansion (**4e**).

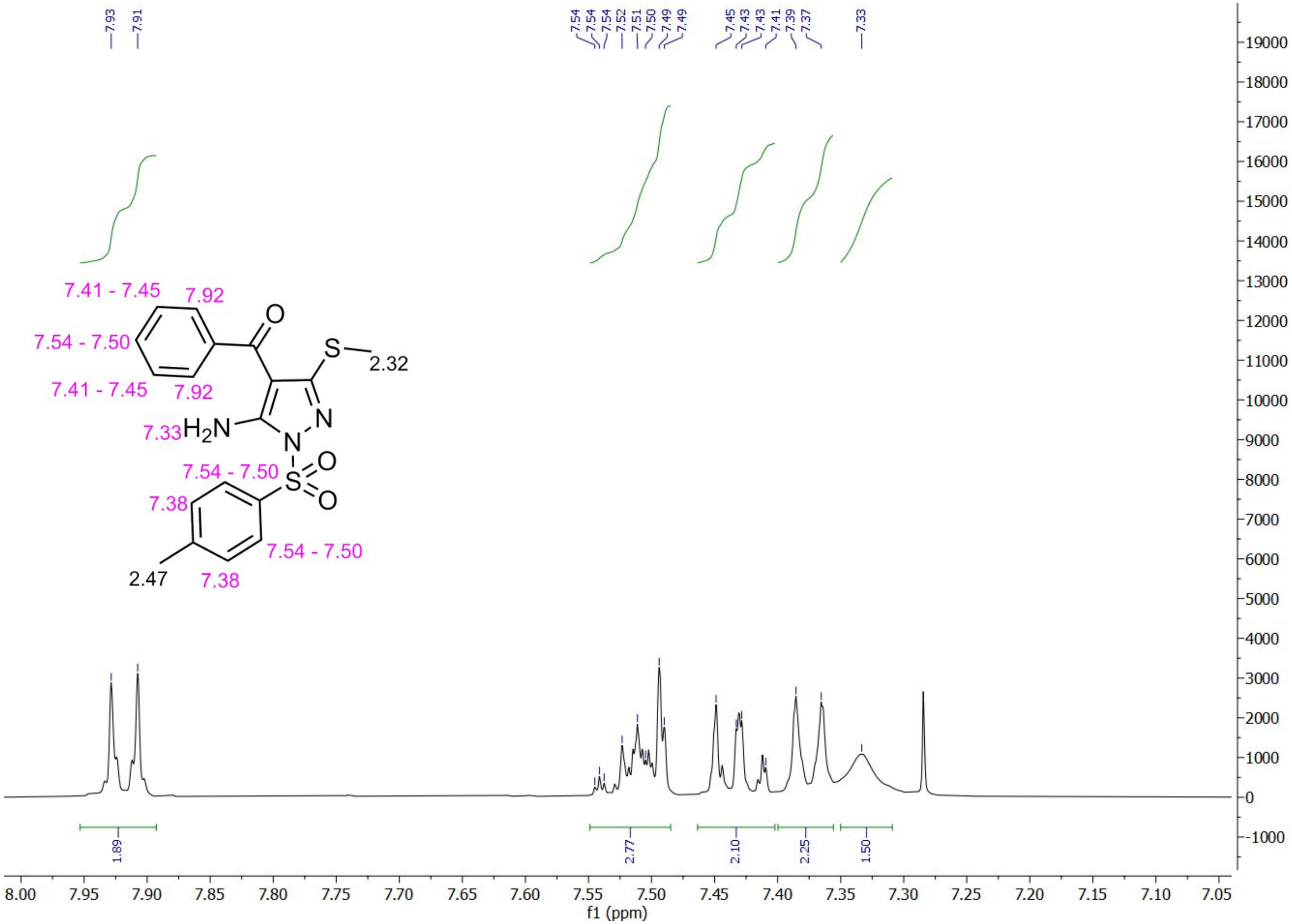

Figura S22. ^{13}C NMR (101 MHz) spectrum of (5-amino-3-(methylthio)-1-tosyl-1H-pyrazol-4-yl)(phenyl)methanone in $CDCl_3$ (**4e**).

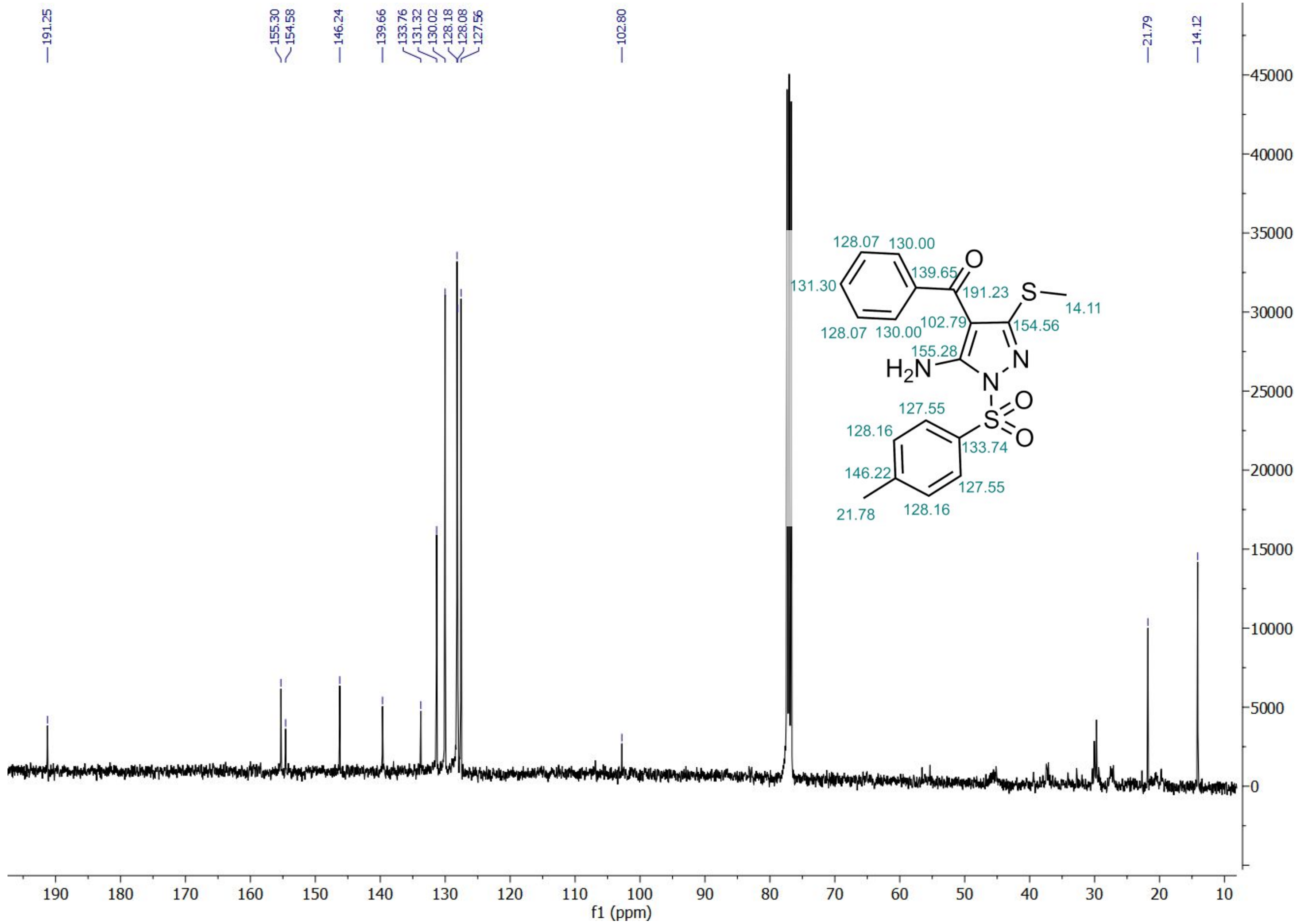

Figura S23. Infrared spectrum of (5-amino-3-(methylthio)-1-tosyl-1H-pyrazol-4-yl)(phenyl)methanone (**4e**).

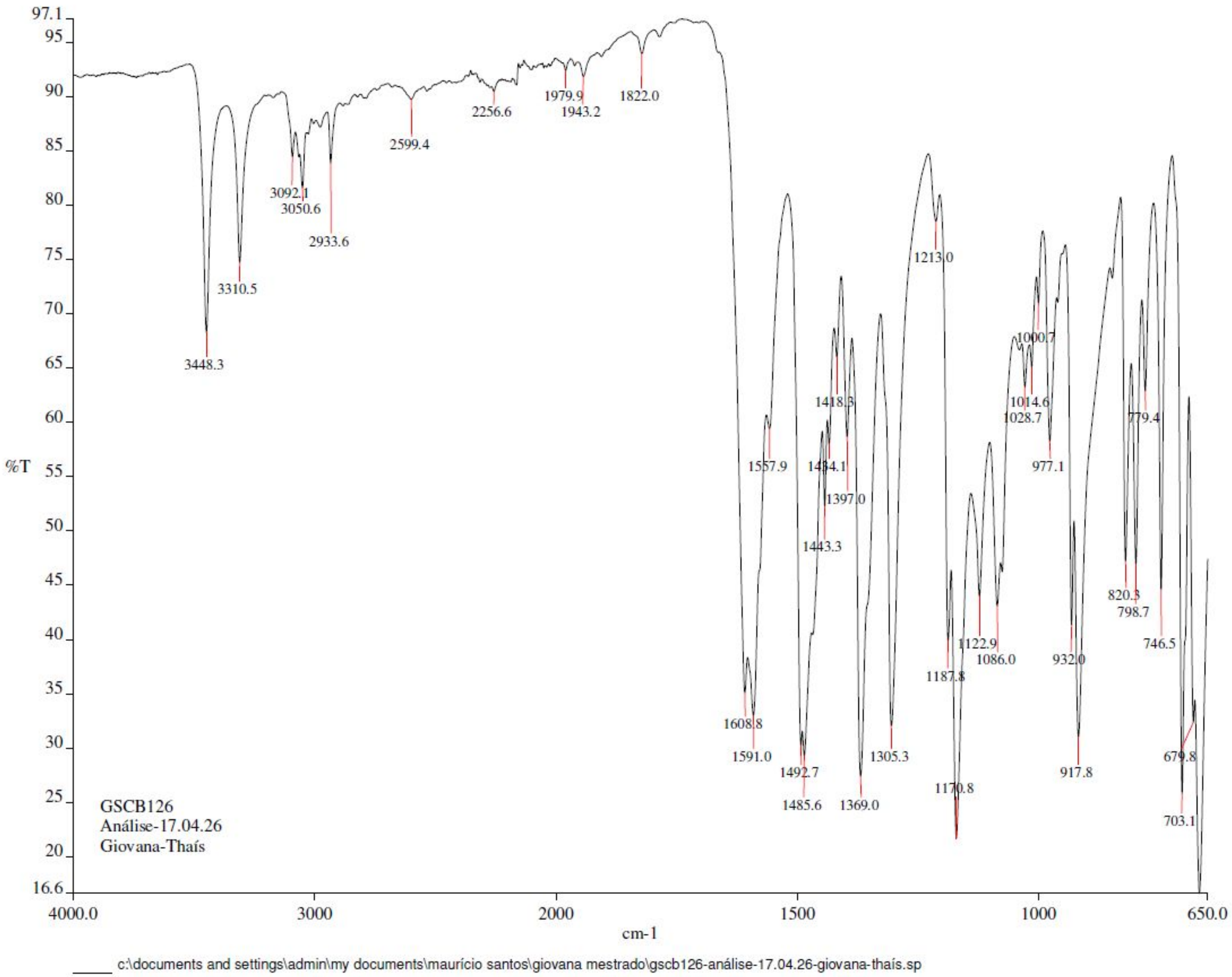

Figura S24. High resolution mass spectrum of (5-amino-3-(methylthio)-1-tosyl-1H-pyrazol-4-yl)(phenyl)methanone (**4e**).

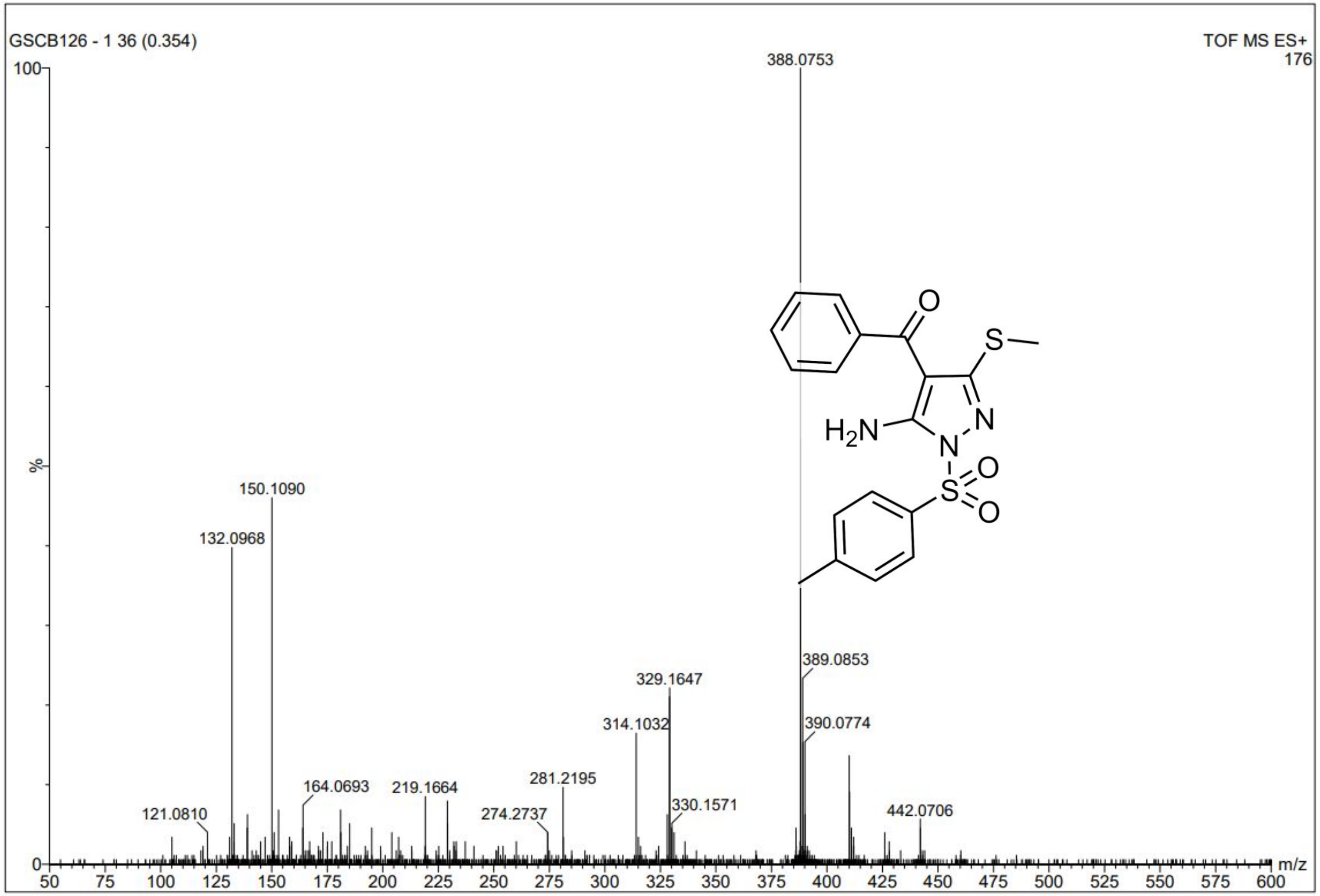

Supplement: Supplementary file 1 [file ao6c06392_si_001.pdf]
